# A seven-helix protein constitutes stress granules crucial for regulating translation during human-to-mosquito transmission of *Plasmodium falciparum*

**DOI:** 10.1371/journal.ppat.1007249

**Published:** 2018-08-22

**Authors:** Sandra Bennink, Andreas von Bohl, Che J. Ngwa, Leonie Henschel, Andrea Kuehn, Nicole Pilch, Tim Weißbach, Alina N. Rosinski, Matthias Scheuermayer, Urska Repnik, Jude M. Przyborski, Allen M. Minns, Lindsey M. Orchard, Gareth Griffiths, Scott E. Lindner, Manuel Llinás, Gabriele Pradel

**Affiliations:** 1 Division of Cellular and Applied Infection Biology, RWTH Aachen University, Aachen, Germany; 2 Research Center for Infectious Diseases, University of Würzburg, Würzburg, Germany; 3 Department of Biosciences, University of Oslo, Oslo, Norway; 4 Division of Parasitology, University of Marburg, Marburg, Germany; 5 Department of Biochemistry and Molecular Biology, The Pennsylvania State University, University Park, PA, United States of America; 6 Department of Chemistry & Huck Center for Malaria Research, The Pennsylvania State University, University Park, PA, United States of America; Weill Medical College of Cornell University, UNITED STATES

## Abstract

The complex life-cycle of the human malaria parasite *Plasmodium falciparum* requires a high degree of tight coordination allowing the parasite to adapt to changing environments. One of the major challenges for the parasite is the human-to-mosquito transmission, which starts with the differentiation of blood stage parasites into the transmissible gametocytes, followed by the rapid conversion of the gametocytes into gametes, once they are taken up by the blood-feeding *Anopheles* vector. In order to pre-adapt to this change of host, the gametocytes store transcripts in stress granules that encode proteins needed for parasite development in the mosquito. Here we report on a novel stress granule component, the seven-helix protein 7-Helix-1. The protein, a homolog of the human stress response regulator LanC-like 2, accumulates in stress granules of female gametocytes and interacts with ribonucleoproteins, such as CITH, DOZI, and PABP1. Malaria parasites lacking 7-Helix-1 are significantly impaired in female gametogenesis and thus transmission to the mosquito. Lack of 7-Helix-1 further leads to a deregulation of components required for protein synthesis. Consistently, inhibitors of translation could mimic the 7-Helix-1 loss-of-function phenotype. 7-Helix-1 forms a complex with the RNA-binding protein Puf2, a translational regulator of the female-specific antigen Pfs25, as well as with *pfs25*-coding mRNA. In accord, gametocytes deficient of 7-Helix-1 exhibit impaired Pfs25 synthesis. Our data demonstrate that 7-Helix-1 constitutes stress granules crucial for regulating the synthesis of proteins needed for life-cycle progression of *Plasmodium* in the mosquito vector.

## Author summary

*Plasmodium falciparum* is a unicellular parasite responsible for the majority of 440,000 deaths due to malaria every year. Malaria is a vector-borne disease, transmitted by blood-feeding *Anopheles* mosquitoes. While the high-replicating red blood cell stages are responsible for the symptoms of malaria, the gametocytes, sexual precursors that form in the blood, are crucial for the transmission of the parasite from the human to the mosquito. Once taken up by the mosquito during blood feeding, the gametocytes rapidly convert to gametes, which mark the mosquito-specific phase of the parasite life-cycle. Due to their essential role for malaria transmission, gametocytes represent prime targets for transmission-blocking strategies intended to prevent spread of the disease. In this study, we explored the signaling pathways leading to gametogenesis and identified a hitherto unknown protein, which structurally belongs to the class of seven-helix proteins. The protein, named 7-Helix-1, is specifically expressed in female gametocytes and disruption of its gene leads to impaired gamete formation and reduced transmission of malaria parasites to mosquitoes. Deletion of 7-Helix-1 caused a significant imbalance of parasite components essential for protein synthesis. We thus propose that 7-Helix-1 is needed to coordinate the increased need of proteins at the onset of gametogenesis.

## Introduction

In eukaryotes, proteins comprising seven helix domains are classified as receptors capable of binding a high variety of ligands. Because many of these receptors transduce signals to heterotrimeric G proteins, they are commonly referred to as G protein-coupled receptors (GPCR) (reviewed in [1]). Alternative names include serpentine receptors (SRs), seven-transmembrane receptors or seven-helix proteins. These proteins are usually integral parts of cell membranes and able to perceive a broad spectrum of stimuli, such as peptides, small molecules or light (reviewed in [1]). Recent studies have further reported on GPCR-like molecules that are not integrated in cell membranes but found in the cytoplasm of a variety of mammalian tissues, such as liver, brain or muscle cells. Due to their homologies with prokaryotic lanthionine cyclases (LanC) of gram-positive bacteria, these GPCR-like proteins were termed LanC-like (LanCL) proteins [2–5].

GPCRs generally transduce the signals via two main pathways, i.e. the cAMP pathway, during which a transmembrane adenylate cyclase is activated, and the phosphatidylinositol pathway, which is mediated by activation of phospholipase PI-PLC, leading to the formation of the second messenger IP_3_ and subsequent increase in intracellular calcium (reviewed in [6,7]). The physiological importance of the GPCR family is evident from its great expansion in the genomes of complex eukaryotes, with typically around 1,000 different members in mammalian species (reviewed in [1]). Noteworthy, GPCRs are involved in many diseases and are the targets of approximately 40% of all currently used drugs (reviewed in [8]).

Components of GPCR signaling cascades have also been identified in *Plasmodium falciparum*, the causative agent of malaria tropica, which is responsible for the majority of the ~440,000 deaths due to malaria per year [9]. Various studies demonstrated that the random contact of *P*. *falciparum* merozoites with red blood cells (RBCs) leads to PI-PLC activation and consequently to a rise in intracellular calcium, causing the discharge of specialized secretory vesicles, the micronemes. This allows secretion of microneme-resident adhesion proteins, which support RBC binding and invasion by the merozoites (reviewed in [10]).

Calcium-dependent signaling cascades are also involved in gametogenesis, which takes place in the midgut of the *Anopheles* vector (reviewed in [11,12]). Following uptake of the transmissible gametocytes by a blood-feeding mosquito, a cGMP-dependent signaling pathway induces IP_3_ synthesis and a rise in cytosolic calcium. The increased calcium levels subsequently activate calcium-dependent protein kinases, which control DNA replication during the formation of male gametes as well as the synthesis of proteins required for life-cycle progression in the mosquito (reviewed in [11–13]). Protein synthesis is particularly important during female gametogenesis, when translational repression of a variety of mRNAs encoding midgut stage-specific proteins, is released. These transcripts are stored in female gametocytes bound to non-translating messenger ribonucleoproteins [14–16]. The complexes of mRNA and ribonucleoproteins resemble stress granules (SGs), i.e. non-membranous cytosolic RNA-protein aggregates, which function in protecting mRNA stalled in translation initiation under cell stress (reviewed in [17–19]). The storage of transcript in female gametocytes is considered a pro-active measure of the parasite to rapidly adapt to the change of host.

While these signaling pathways suggest the involvement of GPCRs in signal perception, neither a heterotrimeric G-protein nor an IP_3_-responsive calcium channel has been identified until now in *Plasmodium* [20,21]. Nonetheless, four potential GPCRs comprising typical 7-helix domains were identified by the *in silico* analysis in the *P*. *falciparum* genome ([22]; reviewed in [23]). The proteins were termed SR1, 10, 12, and 25 according to their homologies with other eukaryotic SR family members. A recent study provided a functional analysis for SR25, which acts as a potassium sensor in blood stage parasites and, upon stimulation, activates PI-PLC leading to increased cytosolic calcium levels [24].

In this study, we have screened the genome of *P*. *falciparum* for additional GPCR-like proteins and identified a novel protein with typical heptahelical motifs. We show that this protein, termed 7-Helix-1, does not represent a transmembrane GPCR, but is linked to translational repressors in the stress granules of female gametocytes, where it is crucial for the regulation of protein synthesis at the onset of gametogenesis.

## Results

### 7-Helix-1 shares homologies with LanC-like protein 2

In order to identify potential heptahelical proteins of gametocytes we searched the *P*. *falciparum* genome for genes with homologies to eukaryotic GPCR. In addition to the genes of the previously described SR1, 10, 12 and 25 [22], we identified a gene encoding a putative GPCR (PF3D7_0525400), henceforth termed 7-Helix-1.

**Fig 1:**
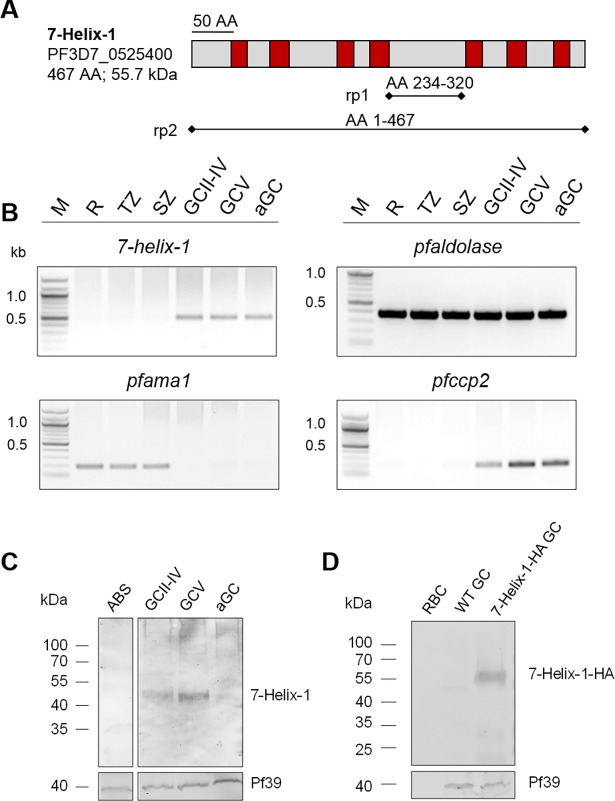
Expression of 7-Helix-1 in *P*. *falciparum* gametocytes. (A) Schematic depicting the domain structure of 7-Helix-1. The seven predicted transmembrane domains (red boxes) are highlighted. The black bars underneath the structure of 7-Helix-1 denote regions of the recombinant proteins. AA, amino acids. (B) Transcription profile of *7-helix-1* in the WT NF54 blood and sexual stages. Transcript of *7-helix-1* (527 bp) was amplified by diagnostic RT-PCR from ring stages (R), trophozoites (TZ), schizonts (SZ), immature (GCII-IV) and mature (GCV) gametocytes and gametocytes at 15 min p.a. (aGC). Transcript analysis of *pfaldolase* (378 bp) was used for loading control. Transcript analyses of *pfama1* (189 bp) and *pfccp2* (198 bp) were used to demonstrate purity of the asexual blood stage and gametocyte samples, respectively. (C) Expression of 7-Helix-1 in the blood and sexual stages. WB analysis of lysates from asexual blood stages (ABS), immature (GCII-IV), mature (GCV) and activated gametocytes (aGC; at 30 min p.a.) of WT NF54 using the mouse anti-7-Helix-1rp2 antisera was employed for detection of 7-Helix-1 (~50 kDa). Equal loading was confirmed using a polyclonal mouse anti-Pf39 antiserum (~39 kDa). (D) Expression of HA-tagged 7-Helix-1 in the 7-Helix-1-HA line. Gametocyte (GC) lysates of WT NF54 and the 7-Helix-1-HA line were subjected to WB and immunolabeled with rabbit anti-HA antibodies to detect 7-Helix-1-HA (~60 kDa). Lysate of uninfected RBCs was used for negative control; equal loading was confirmed using a polyclonal mouse anti-Pf39 antiserum (~39 kDa). Results (in B-D) are representative of two to three independent experiments.

The gene *7-helix-1* encodes a 55.7 kDa protein that comprises seven predicted transmembrane domains (Fig 1A). Proteins homologous to 7-Helix-1 are encoded in the genomes of several other organisms, e.g. the *Plasmodium* species *P*. *vivax* and *P*. *berghei* (putative GPCRs comprising a LanCL domain), *Homo sapiens* (hLanCL1 and hLanCL2), *Mus musculus* (mLanCL1 and mLanCL2) and *Arabidopsis thaliana* (LanCL protein GCR2) [25–28]. All of them share seven conserved GXXG motifs [28], with 7-Helix-1 being the only exception, since the protein only shares six of the motifs, while the seventh is changed to GXXA instead (S1 Fig). Furthermore, the repeats include the highly conserved HG motif in repeat four, WCXG in repeat five and CHG in repeat six that are critical for the enzymatic activity of the LanCL proteins [29,30]. The highest homology, i.e. 38%, was found between 7-Helix-1 and hLanCL2, an intracellular abscisic acid receptor of human liver and muscle cells [2,3,5,31,32].

Phylogenetic analysis of 7-Helix-1 and related proteins from other organisms revealed that 7-Helix-1 forms a distinct cluster with the homologous plasmodial proteins (S2A Fig). The 3D structure of 7-Helix-1 was modelled using the I-TASSER server based on the crystal structure of hLanCL1 [27] and showed that 7-Helix-1 comprises a total of 14 α-helices that form two layers of α-helical barrels with seven helices each (S2B Fig). The combined data indicate that 7-Helix-1 is not a typical membrane-spanning GPCR-like receptor, but is rather related to the cytosolic hLanCL proteins.

### 7-Helix-1 localizes to granules in female gametocytes

Diagnostic RT-PCR was employed to investigate expression of *7-helix-1* in the *P*. *falciparum* blood stages and demonstrated similar transcript levels in immature (stage II-IV) and mature (stage V) gametocytes as well as gametocytes at 15’ post-activation (p.a.). In contrast, no expression was detected in the asexual blood stages (Fig 1B). Transcript analysis of the housekeeping gene *pfaldolase* was used as a loading control, and purity of the asexual blood stage and gametocyte samples was demonstrated by amplification of transcripts for the asexual blood stage-specific gene *pfama1* (apical membrane antigen 1) [33] and for the gametocyte-specific gene *pfccp2* (LCCL-domain protein 2) [34] (Fig 1B).

Mouse antisera were generated against two recombinant 7-Helix-1 peptides (7-Helix-1rp1 and 7-Helix-1rp2; see Fig 1A) to be used for protein expression analysis. Lysates of mixed asexual blood stages, immature gametocytes (stage II-IV), mature gametocytes (stage V) and gametocytes at 30’ p.a. were subjected to Western blot (WB) analysis. Immunoblotting with 7-Helix-1rp2 antisera demonstrated 7-Helix-1 migrating at a molecular weight of ~ 50 kDa in the lysates of immature and mature gametocytes, but detected no signal in asexual blood stage lysates (Fig 1C). Only a weak protein band was detected in the lysates of activated gametocytes. Immunoblotting with mouse antiserum directed against the endoplasmic reticulum-resident protein Pf39 was used as a loading control (Fig 1C).

**Fig 2:**
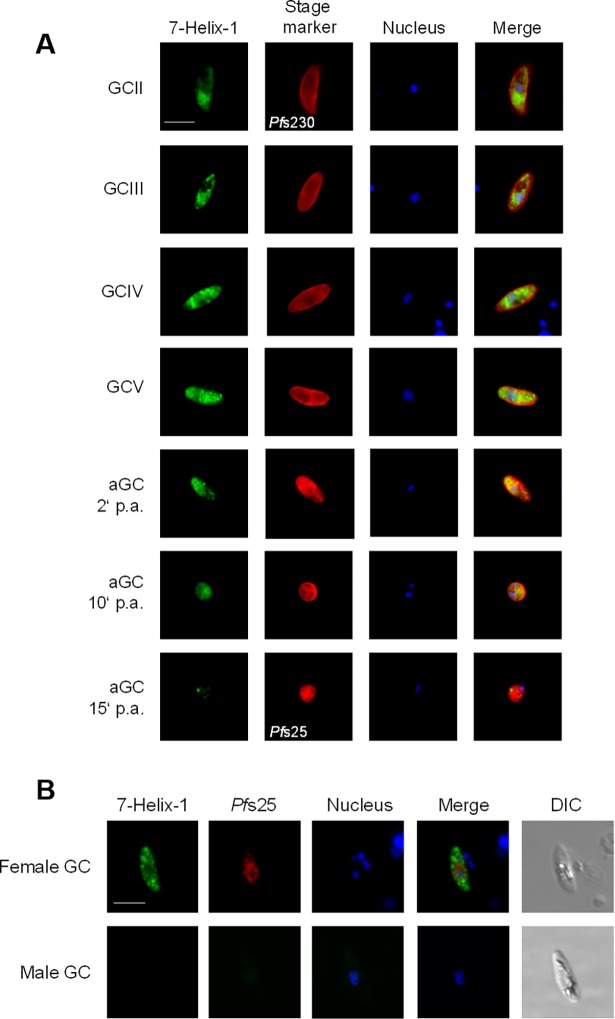
Granular localization of 7-Helix-1 in female *P*. *falciparum* gametocytes. (A) Immunolocalization of 7-Helix-1 in the sexual stages. Gametocytes (GC stage II–V) and activated gametocytes (aGC; at 2, 10 and 15 min p.a.) of WT NF54 were immunolabeled with mouse anti-7-Helix-1rp1 antisera (green) and counterlabeled with rabbit antibodies directed against Pfs230 and Pfs25 as indicated (red). (B) Female-specific expression of 7-Helix-1. WT NF54 gametocytes were immunolabeled with mouse anti-7-Helix-1rp1 antisera (green) and counterlabeled with anti-Pfs25 antisera (red). Nuclei (in A and B) were highlighted by Hoechst33342 nuclear stain (blue). Bar, 5 μm. Results are representative of five independent experiments.

Indirect immunofluorescence assays (IFAs), using either of the two antisera, revealed that 7-Helix-1 was abundantly expressed in immature and mature gametocytes, where it localized within cytosolic granules (Fig 2A; S3 Fig). Upon activation of the gametocytes, 7-Helix-1 expression decreased within 15 min. In the asexual blood stages, 7-Helix-1 was absent (S4A and S4B Fig). When sera from non-immunized mice (NMS) was used in the IFAs as a negative control, no labelling was detectable in either asexual blood stages or gametocytes (S5 Fig).

We also generated a transfectant line, expressing 7-Helix-1 tagged to hemagglutinin (HA) and streptavidin (Strep), (S6A Fig). Vector integration into the 3´-end of the *7-helix-1* locus was demonstrated by diagnostic PCR (S6B Fig). Subsequent WB, using an anti-HA antibody, demonstrated the expression of the 7-Helix-1 protein fused to HA and Strep (termed 7-Helix-1-HA), migrating at the expected molecular weight of ~60 kDa (Fig 1D). No protein band was detected, though, in lysates of wildtype (WT) gametocytes or in non-infected RBCs, used for negative control. IFAs confirmed that 7-Helix-1-HA is expressed in gametocytes and here accumulates in granular structures, while no labeling was detected in WT gametocytes (S6C Fig).

To verify the cytosolic localization of 7-Helix-1 we used gametocytes of the 7-Helix-1-HA line for subcellular fractioning. Subsequent WB using an anti-HA antibody detected 7-Helix-1-HA in the soluble protein fraction, but not in the integral and insoluble protein fractions (S7A Fig). Immunoblotting with antisera against the cytosolic falcilysin [35], the peripheral PfCCp2 and the parasitophorous vacuolar membrane-resident Pfs16 [36] were used as controls.

7-Helix-1 was found only in a subpopulation of gametocytes, when these were identified via immunolabelling for Pfs230, a plasmalemma-associated protein of the cysteine-rich superfamily, which is present in both male and female gametocytes (reviewed in [37,38]). Counterstaining of gametocytes by immunolabelling of Pfs25, a female-specific EGF domain protein (reviewed in [38]), revealed that 7-Helix-1 is expressed in 94.9 ± 2.43% of Pfs25-positive gametocytes, while only 78.9 ± 1.97% of Pfs230-positive cells were positive for 7-Helix-1 labelling (n = 100, in ten replicates). Pfs25-negative gametocytes (100%) were also negative for 7-Helix-1 labelling (Fig 2B). Taking into account, that the ratio of male:female gametocytes is female-biased with one male for about five female gametocytes (reviewed in [11]), these findings point to a female-specific expression of 7-Helix-1 in *P*. *falciparum* and are in accord with a reported female-specific expression by transcriptomics studies [39]. Noteworthy, 7-Helix-1 did not co-localize with the female-specific protein Pfg377, a marker for osmiophilic bodies [40], indicating that 7-Helix-1 is not present in these vesicles (S7B Fig). In conclusion, 7-Helix-1 is specifically expressed in female gametocytes and here localizes in cytoplasmic granules.

### Parasites lacking 7-Helix-1 are impaired in female gametogenesis

For functional analysis, the WT *7-helix-1* locus was disrupted via single cross-over homologous recombination (S8A Fig). Two clonal lines (1D12 and 2E6) were isolated and successful integration of the plasmid into the genome was verified by diagnostic PCR (S8B Fig). Vector integration was further validated by sequencing of the integration locus for clone 2E6 (S9 Fig). The absence of the protein in 7-Helix-1-KO gametocytes of both lines compared to WT gametocytes was subsequently demonstrated by IFA (S8C Fig). Similarly, WB could detect no 50-kDa protein in gametocyte lysates of the two 7-Helix-1-KO lines compared to WT (S8D Fig).

Subsequent phenotype analyses showed that the morphology and intraerythrocytic development of the asexual blood stages of the two 7-Helix-1-KO lines was normal compared to WT (S10A and S10B Fig). Furthermore, morphology and development of gametocytes during maturation from stage II to V was comparable to WT (S11A and S11B Fig). In addition, the percentage of female gametocytes did not differ in the 7-Helix-1-KO compared to the WT (89.3 ± 1.53% and 86.0 ± 3.46%, respectively; two independent experiments performed in triplicate), as shown by immunolabelling with anti-7-Helix-1-rp1 antisera.

**Fig 3:**
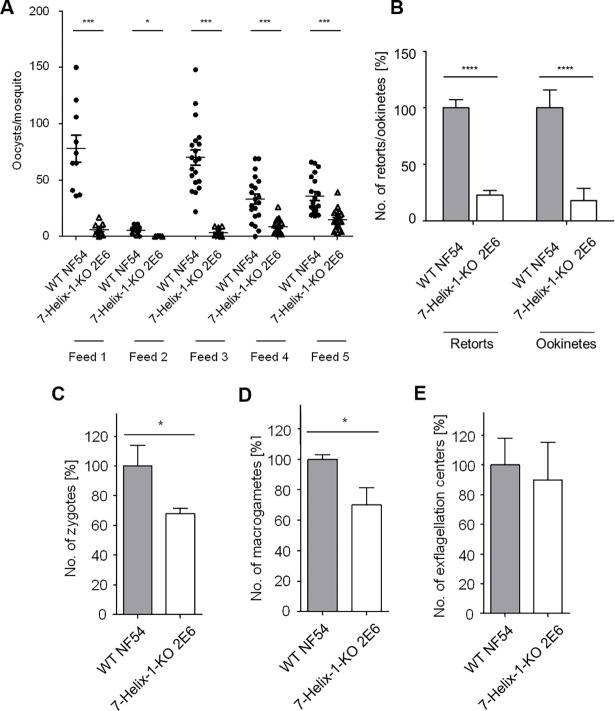
Impaired transmission of 7-Helix-1-KO gametocytes to mosquitoes. (A) Mosquito infection efficiency of 7-Helix-1-KO. Enriched mature gametocytes of WT NF54 or the 7-Helix-KO line 2E6 were fed to *An*. *stephensi* mosquitoes via SMFAs. The numbers of oocysts per midgut were counted at day 10 p.i. in five independent feeds. * p ≤ 0.05; *** p ≤ 0.001 (Mann-Whitney-U test). (B) Efficiency of 7-Helix-1-KO to develop into mosquito midgut stages. Following SMFAs as described above, midgut smears at 24 h p.i. were subjected to IFA, the ookinetes and retorts were immunolabeled with rabbit anti-Pfs28 antisera and counted in 30 optical fields for four times (mean ± SD). (C-E) Efficiency of 7-Helix-1-KO to undergo sexual reproduction. Mature WT NF54 and 7-Helix-1-KO 2E6 gametocytes were activated *in vitro*. Samples were taken at 4 h p.a. (zygotes), 30 min p.a. (macrogametes) or 15 min p.a. (exflagellation centers). Zygotes (C) and macrogametes (D) were subjected to IFA for immunolabelling with anti-Pfs25 antibody and counted in 30 optical fields in triplicate; exflagellation centers (E) were counted in 30 optical fields for four times using light microscopy (mean ± SD). The numbers of WT parasites were set to 100% (for B-E). * p ≤ 0.05; **** p ≤ 0.0001 (Student‘s *t*-test). Results (in B-E) are representative of three to eight independent experiments.

When mature gametocytes of the 7-Helix-1-KO line 2E6 were fed to *Anopheles stephensi* mosquitoes via standard membrane feeding assays (SMFAs), the number of salivary gland sporozoites at day 17 post-infection (p.i.) was reduced to 8.0% and 9.0% compared to the numbers of the WT control in two independent experiments (S1 Table). Further, at day 10 p.i. the numbers of oocysts attached to the mosquito midgut were significantly reduced compared to the WT (Fig 3A). The numbers of retorts and ookinetes were quantified in midgut smears at 24 h p.i. via immunolabelling with rabbit antisera against Pfs28. Quantification revealed a significant reduction of retorts (22.7 ± 3.98%) and ookinetes (17.7 ± 10.88%) when the mosquitoes were fed with the 7-Helix-1-KO line 2E6 compared to WT control (set to 100%) (Fig 3B). Furthermore, we detected a significant decrease in the numbers of zygotes (67.8 ± 3.70%), and macrogametes (69.9 ± 11.21%) formed by the 7-Helix-1-KO line 2E6 compared to WT, as investigated via immunolabelling with anti-Pfs28 and anti-Pfs25 antibody at 4 h and 30 min p.a. *in vitro*, respectively (Fig 3C and 3D). No significant differences were observed between 7-Helix-1-KO 2E6 and WT gametocytes in the ability to form motile male microgametes (termed exflagellation) upon activation (89.7 ± 25.54%) (Fig 3E).

**Fig 4:**
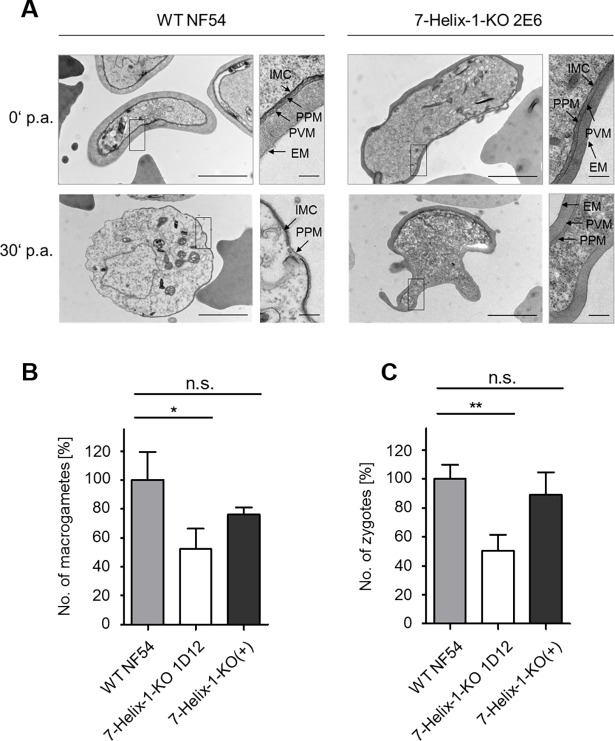
Ultrastructural analysis of activated 7-Helix-1-KO gametocytes and phenotype rescue. (A) Ultrastructure of 7-Helix-1-KO gametocytes. Transmission electron microscopic analyses of WT NF54 and 7-Helix-1-KO 2E6 gametocytes at 0 and 30 min p.a.. EM, erythrocyte membrane; IMC, inner membrane complex; PPM, parasite plasma membrane; PVM, parasitophorous vacuole membrane. Bar, 2 μm; enlargement, 0.2 μm. (B, C) 7-Helix-1-KO phenotype rescue by episomal complementation. Gametocytes of WT NF54, 7-Helix-1-KO 1D12 and 7-Helix-1-KO(+) were activated *in vitro*. Samples were taken at 30 min (macrogametes, B) and 4 h (zygotes, C) p.a. and immunolabeled with anti-Pfs25 antibody. The numbers of parasites were counted in 30 optical fields in triplicate (mean ± SD). The numbers of WT parasites were set to 100%. n.s., not significant; * p ≤ 0.05; ** p ≤ 0.01 (One-Way ANOVA with Post-Hoc Bonferroni Multiple Comparison test). Results (in B and C) are representative of three independent experiments.

On the ultrastructural level, non-activated 7-Helix-1-KO 2E6 gametocytes exhibited the typical morphology described for WT gametocytes, having a prominent enveloping erythrocyte membrane (EM) and parasitophorous vacuole membrane (PVM) as well as the inner membrane complex (IMC) (Fig 4A). At 30 min p.a., the WT macrogametes have egressed from the RBC, and at this time point, the PVM has fully ruptured, while the IMC has partially disintegrated. These morphological changes upon gametocyte activation are in accord with previous reports [41]. In 7-Helix-1-KO gametocytes at 30 min p.a., however, approximately 39% of the parasites had not rounded up (30 ultrasections investigated). The PVM and EM were still present in 39% and 69% of the activated 7-Helix-1-KO gametocytes, respectively, while in 46% of the parasites, the IMC was still intact (Fig 4A).

A complementation line was generated by transfecting the 7-Helix-1-KO line 1D12 with an episomal copy of the gene (fused to a GFP-encoding sequence) under the *fnpa* promotor (a LCCL-domain protein). The presence of the vector in the 7-Helix-1-KO(+) line was confirmed via diagnostic PCR (S12A Fig). Amplification of *pfama1* was used as a positive control in the PCRs. Furthermore, WB, using the mouse anti-7-Helix-1rp2 antisera, detected a band at ~ 75 kDa in the 7-Helix-1-KO(+) lysate, corresponding to the 7-Helix-1-GFP fusion protein (S12B Fig). A similar band was detected, when a rabbit anti-GFP antibody was used for immunoblotting. Controls included the detection of 7-Helix-1 in the WT lysate migrating at ~ 50 kDa, while no corresponding band was detected in the 7-Helix-1-KO 1D12 lysate. Immunoblotting with mouse anti-Pf39 antiserum was used as a loading control (S12B Fig). Protein expression and granular localization of the 7-Helix-1-GFP fusion protein in 7-Helix-1-KO(+) gametocytes was further verified via IFA, using either of the two generated anti-7-Helix-1 antisera (S12C and S12D Fig).

The 7-Helix-1-KO(+) line was used to conduct phenotype rescue experiments. While in the 7-Helix-1-KO line 1D12, the numbers of macrogametes (52.2 ± 14.17%) and zygotes (50.2 ± 11.32%) were significantly reduced compared to WT (set to 100%), the 7-Helix-1-KO(+) line exhibited higher numbers of macrogametes (76.1 ± 4.75%) and zygotes (89.2 ± 15.30%). These numbers did not differ significantly from the numbers of macrogametes and zygotes in activated WT parasites (Fig 4B and 4C). Our combined data demonstrate that the loss of 7-Helix-1 results in severely impaired female gametogenesis.

### The loss of 7-Helix-1 results in the deregulation of genes required for translation

To investigate potential transcriptional changes caused by the lack of 7-Helix-1, we employed microarray analyses. Gametocytes at 30 min p.a. of the WT and the 7-Helix-1-KO line 2E6 were purified, the cDNA was synthesized and analyzed using a *P*. *falciparum* DNA Agilent microarray chip containing DNA spots corresponding to the 5,363 coding genes of nucleus, mitochondrion and apicoplast [42,43] (S1 File).

In the 7-Helix-1-KO line 2E6, 198 genes were identified with transcript levels more than 1.5-fold higher compared to WT; 59 of these genes exhibited factors of 2 or higher (S2 File). Gene ontology (GO) enrichment analyses ranked the majority of transcriptionally up-regulated genes to the biological processes of translation and biosynthesis with a high number of gene products being components of ribosomes (S3 File). Further, 122 genes of the 7-Helix-1-KO exhibited more than 1.5-fold lower transcript levels compared to WT. Of these, 34 exhibited factors of 0.5 or lower and included the gene PF3D7_0525400 encoding 7-Helix-1 (down-regulation factor: 0.19). Noteworthy, 60 of the transcriptionally down-regulated genes encrypt non-coding RNAs (S2 File).

**Fig 5:**
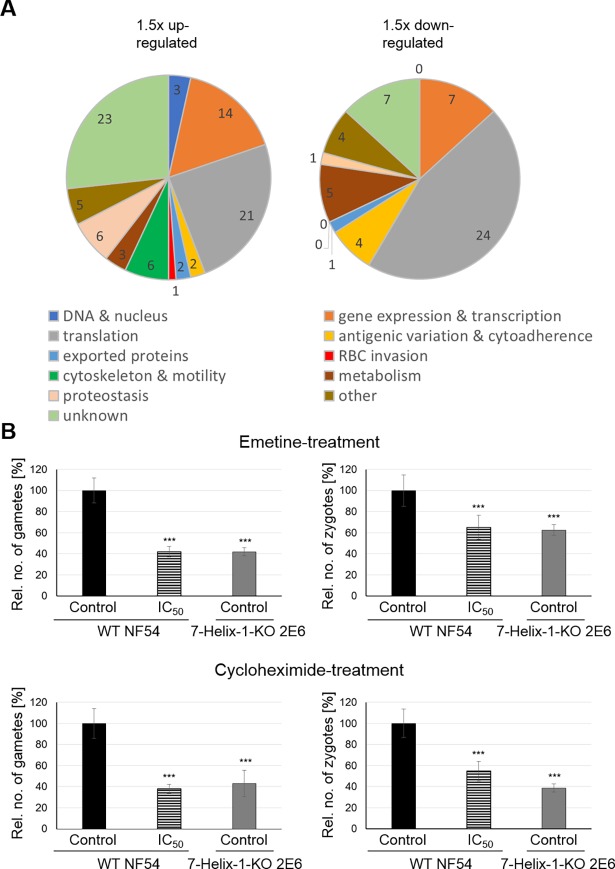
Transcriptional deregulation in 7-Helix-1-KO gametocytes and loss-of-function mimicry by chemical inhibition of translation. (A) Pie charts depicting genes with deregulated transcripts in activated 7-Helix-1-KO gametocytes grouped by function. Mature gametocytes of strain WT NF54 and 7-Helix-1-KO 2E6 were activated and samples were collected at 30 min p.a. Total RNA was isolated and cDNA synthesized to be employed in microarray assays. Genes expressed in gametocytes (defined by transcript levels > 50% of peak transcript levels) with relative transcript levels greater (left pie) and lower (right pie) than 1.5-fold were grouped based on the predicted functions. Microarray analyses were performed once. (B) Loss-of-function analysis in gametocytes following chemical inhibition of translation. Mature gametocytes of WT NF54 or 7-Helix-1-KO line 2E6 were treated with emetine or cycloheximide at IC_50_ concentrations (as determined by Malstat assay) or solvent alone for 30 min at 37°C, followed by *in vitro* activation. Samples were taken at 30 min (macrogametes) and 4 h (zygotes) p.a. and immunolabeled with anti-Pfs25 antibody. The numbers of parasites per 1,000 RBCs were counted five times (mean ± SD). Gametes/zygotes formed in the solvent-treated WT line were set to 100%. *** p ≤ 0.001 (One-Way ANOVA with Post-Hoc Bonferroni Multiple Comparison test). Results are representative for two independent experiments.

For in-depth analyses, we focused on transcriptionally deregulated genes highly expressed in gametocytes and grouped these according to their predicted functions. We identified 86 genes that were transcriptionally up-regulated in activated 7-Helix-1-KO gametocytes (S2 File). The majority of the genes could be assigned to functions in transcription and translation (Fig 5A). Genes assigned to transcription included the RNA-binding proteins ALBA1 and ALBA4 [44,45] as well as PF3D7_0823200, all of which are components of the mRNA-bound proteome of *P*. *falciparum* [46]. Out of the 21 genes assigned to translation, 19 coded for ribosomal proteins as part of the 60S or 40S subunits (S2 File). Further, two genes encode for putative elongation initiation factors, i.e. eIF5A (PF3D7_0913200) and SUI1 (PF3D7_1243600). In addition, 53 genes were transcriptionally down-regulated in the activated 7-Helix-1-KO gametocytes, which were mainly associated with translation. Out of 24 genes linked to translation, 10 genes encode tRNAs and 12 genes code for small nucleolar RNAs needed for the assembly of the 60S and 40S ribosomal subunits (S2 File). In addition, a high proportion of genes with deregulated transcription levels were of unknown function (Fig 5A).

In order to validate the microarray data, we randomly chose seven of the identified genes (five of them transcriptionally up-regulated and two down-regulated in microarray analysis) and analyzed differences in transcript expression via real time RT-PCR. For this, gametocytes of the 7-Helix-1-KO line 2E6 and WT gametocytes were purified at 30 min p.a. and total RNA was isolated followed by cDNA synthesis. Real time RT-PCR was performed and the threshold cycle number (Ct) calculated, which was normalized to the Ct of the endogenous control gene encoding *P*. *falciparum* seryl tRNA-ligase (PF3D7_0717700) as reference. Fold changes in the 7-Helix-1-KO in relation to the WT were determined. The five genes that were more than 2-fold up-regulated in activated 7-Helix-1-KO gametocytes in microarray analysis also showed transcript up-regulation by 2-fold or more in real time RT-PCR (S13A Fig). Further, in the absence of 7-Helix-1, the two genes that exhibited a more than 2-fold down-regulation in microarray studies also had decreased transcript levels. In addition, potential changes in the transcript expression of SR1, SR10, SR12 and SR25 were investigated in mature and activated gametocytes, however, no significant changes were detected (S13B Fig). In conclusion, the transcriptomics data point to an imbalance of components required for protein synthesis in gametocytes lacking 7-Helix-1.

### Inhibitors of translation mimic the 7-Helix-1 loss-of-function phenotype

To further investigate the potential link between 7-Helix-1 and translation during female gametogenesis, we treated mature WT gametocytes with translation inhibitors and investigated their ability to form macrogametes and zygotes. The translation inhibitors used in the experiments included emetine, which blocks translation by binding the 40S ribosomal subunit [47], and cycloheximide, which inhibits ribosome translocation [48]. Antimalarial concentrations were determined by Malstat assay and revealed IC_50_ values of 35 nM ± 1.4 nM and 435 nM ± 27.4 nM for emetine and cycloheximide, respectively. Chloroquine was used as a positive control in the assays and resulted in growth inhibition with a mean IC_50_ value of 22 nM ± 2.8 nM.

Mature gametocytes of the WT and the 7-Helix-1-KO line 2E6 were purified, treated with the solvent alone or with the inhibitors at IC_50_ concentration for 30 min, followed by activation. The numbers of macrogametes and zygotes were determined via IFA at 30 min and 4 h p.a., respectively, by immunolabelling with rabbit anti-Pfs25 antisera. Emetine at IC_50_ concentrations resulted in significantly decreased numbers of macrogametes (41.8 ± 4.98%) compared to the solvent control (set to 100%) (Fig 5B). These numbers were comparable to the ones of the 7-Helix-1-KO line 2E6, when treated with the solvent control (41.8 ± 3.80%). Similar results were obtained, when the numbers of zygotes were determined at 4 h p.a. following emetine-treatment. Emetine-treated WT parasites formed significantly less zygotes than the solvent-treated control (65.1 ± 11.32%; WT solvent-control set to 100%). Solvent-treated 7-Helix-1-KO 2E6 parasites exhibited zygote numbers (62.4 ± 5.16%) comparable to emetine-treated WT gametocytes (Fig 5B).

Likewise, WT parasites treated with cycloheximide formed significantly reduced numbers of macrogametes (37.9 ± 4.40%) compared to the solvent control (set to 100%), which were comparable to the numbers of macrogametes in solvent control-treated 7-Helix-1-KO 2E6 parasites (43.2 ± 12.56%) (Fig 5B). Cycloheximide-treatment of WT parasites led to reduced zygote numbers (54.7 ± 9.20%) compared to the solvent control (set to 100%). The numbers of zygotes in the solvent-treated 7-Helix-1-KO 2E6 sample were comparable to the numbers of zygotes in cycloheximide-treated WT parasites (38.7 ± 3.95%) (Fig 5B). In conclusion, the chemical-KO phenotype caused by translational inhibitors resembles the one observed for 7-Helix-1-KO parasites.

### 7-Helix-1 is a component of stress granules

**Fig 6:**
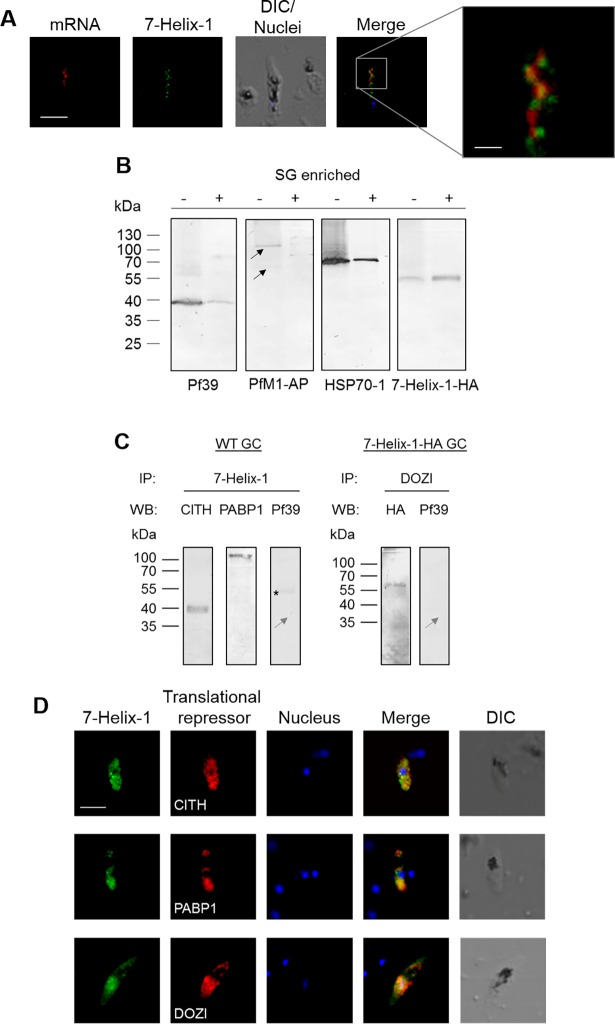
Localization of 7-Helix-1 in SGs and interaction with translational repressors. (A) Co-localization of 7-Helix-1 with mRNA-aggregates. Mature WF NF54 gametocytes were subjected to mRNA-FISH-IFA and mRNA was labeled with a biotinylated oligo-dT25 probe (red); counterlabeling was performed using mouse anti-7-Helix-1rp2 antisera (green). Nuclei were highlighted by Hoechst33342 nuclear stain (blue). Frame indicates the area chosen for enlargement. DIC, differential interference contrast. Bar, 5 μm; enlargement, 1 μm. (B) Accumulation of 7-Helix-1 in SG fractions. Gametocytes of line 7-Helix-1-HA were stressed by treatment with sodium arsenite for 1 h and a SG core fraction enrichment was conducted. Lysates of 7-Helix-1-HA gametocytes (-) and of enriched SGs (+) were subjected to WB, using mouse antisera directed against Pf39 (~39 kDa) or PfM1-AP (~126 and 68 kDa, black arrows) or rabbit antibodies against HSP70-1 (~70 kDa) or the HA-tag to detect 7-Helix-1-HA (~60 kDa). (C) Co-immunoprecipitation of 7-Helix-1 with CITH, PABP1 and DOZI. Lysates of WT NF54 or 7-Helix-1-HA gametocytes were subjected to co-immunoprecipitation assays using polyclonal mouse anti-7-Helix-1rp2 antisera or polyclonal rabbit anti-DOZI antisera, followed by WB using rabbit anti-CITH, anti-PABP1 and anti-HA antibodies or mouse anti-Pf39 antibody to detect precipitated proteins. Grey arrow indicates the expected running line in the negative control. Asterisk indicates a band corresponding to the precipitation antibody. (D) Co-localization of 7-Helix-1 with CITH, PABP1 and DOZI. WT NF54 gametocytes were immunolabeled with mouse anti-7-Helix-1rp2 antisera (green) and rabbit anti-CITH, anti-PABP1, or anti-DOZI antibodies (red). Nuclei were highlighted by Hoechst33342 nuclear stain (blue). DIC, differential interference contrast. Bar, 5 μm. Results are representative of three independent experiments.

The granular localization of 7-Helix-1 in female gametocytes and its crucial role during female gametogenesis let us to investigate a potential link between 7-Helix-1 and SGs. Firstly, an mRNA-detecting fluorescence in situ hybridization experiment coupled with an IFA (mRNA-FISH-IFA) was conducted. A biotinylated poly-dT oligonucleotide was used to bind the poly-A-tails of mRNA aggregates. Poly-dT-oligonucleotide binding was demonstrated with fluorescently labeled Strep, 7-Helix-1 was immunolabeled with anti-7-Helix-1rp2 antisera. The IFA demonstrated the presence of mRNA aggregates in most of the 7-Helix-1-positive granules, indicating that 7-Helix-1 accumulates in RNA dense granules (Fig 6A). A Pearson’s correlation coefficient (-1 for perfect negative correlation, 0 for no correlation, +1 for perfect positive correlation) of 0.79 ± 0.079 has been calculated using ImageJ (n = 15). For control, RNA-FISH-IFAs were performed omitting the poly-dT-oligonucleotide, and no mRNA labeling was observed (S14A Fig). Similar mRNA-FISH-IFA experiments were performed, using the 7-Helix-1-HA line, again demonstrating a co-localization of mRNA aggregates and 7-Helix-1, which was detected using anti-HA antibody (Pearson’s correlation coefficient = 0.67 ± 0.127; n = 15; S14B Fig). For control, a *P*. *falciparum* line RNF1-HA was used, which expresses the HA-tagged ring finger protein RNF1 in gametocytes and which was generated using a similar strategy. No co-labeling of mRNA aggregates and RNF1 was detected (S14C Fig). When anti-Pfs230 antisera was used as a control in either of the mRNA-FISH-IFA experiments, no co-labeling of the protein with mRNA aggregates was detected (S14D and S14E Fig).

The association of 7-Helix-1 with SGs was subsequently confirmed by SG core fraction enrichment. Mature gametocytes of line 7-Helix-1-HA were treated with sodium arsenite to induce stress in these cells and the SGs were isolated by enrichment. Lysates of total mature gametocytes and of the enriched SG fraction were immunoblotted with anti-HA antibody. A prominent 7-Helix-1 band was observed in the SG fraction (Fig 6B). For positive control, rabbit antisera against plasmodial HSP70-1 was used [49], as heat shock proteins are known components of SGs (reviewed in [17]). Accordingly, prominent HSP70-1-positive protein bands, migrating at the expected molecular weight of 70 kDa, were detected in the gametocyte lysate and the SG fraction. Immunoblotting with anti-Pf39 antisera, used as a negative control, labeled Pf39 in the gametocyte lysate, while only minor labeling was detected in the enriched SG fraction. As an additional negative control, the samples were blotted with mouse antisera directed against the full-length and processed form (126 and 68 kDa) of the M1-family alanyl aminopeptidase PfM1-AP [50] and both protein bands could be detected in the gametocyte lysate, but not in the SG fraction (Fig 6B). The combined data show that 7-Helix-1 is enriched in SGs of gametocytes.

Lastly, the interaction of 7-Helix-1 with the known translational repressors CITH (CAR-I/trailer hitch homolog), PABP1 (polyadenylate-binding protein 1) and DOZI (development of zygote inhibited) as components of the female gametocyte-specific SGs [15,45] was investigated via co-immunoprecipitation assay and IFA. When the 7-Helix-1rp2 antisera was used for precipitation, subsequent immunoblotting demonstrated CITH and PABP1 (39 and 97 kDa, respectively) in the precipitate, using the respective antibodies (Fig 6C). When the anti-DOZI antisera was used for precipitation in 7-Helix-1-HA gametocytes, 7-Helix-1-HA (~60 kDa) was detected in the precipitate using the anti-HA antibody for immunoblotting. No Pf39, used for negative control, could be precipitated in either immunoprecipitation. IFA analysis confirmed a co-localization of 7-Helix-1 with CITH, PABP1 and DOZI in female gametocytes (Fig 6D).

### The loss of 7-Helix-1 results in impaired synthesis of translationally repressed Pfs25

In a last set of experiments, we aimed at demonstrating a potential involvement of 7-Helix-1 in translational repression by studying Pfs25, a protein well known to be translationally repressed in the SGs of female gametocytes [16]. Once translational repression is released at the onset of female gametogenesis, Pfs25 is transported to the macrogamete surface, where it plays a role during fertilization (reviewed in [12,37,38]).

In *P*. *falciparum*, the RNA-binding protein Pumilio-2 (Puf2) is involved in translational repression of Pfs25 by binding to the 5´-untranslated region of the *pfs25* gene [16,51]. We thus generated mouse antisera directed against Puf2 via immunization of the mice with a bacterially expressed peptide (S15A Fig). IFAs demonstrated the presence of Puf2 in the cytosol of mature gametocytes, but not in asexual blood stage parasites (S15B Fig), confirming previously published Puf2 expression data [16]. Co-labeling with antibodies directed against the female-specific antigen Pfs25 shows that Puf2 is expressed in gametocytes of both sexes (S15C Fig). Puf2 was also detected, when gametocytes of the 7-Helix-1-KO line 2E6 were immunolabeled with anti-Puf2 antisera (S15D Fig), demonstrating that lack of 7-Helix-1 does not result in loss of Puf2.

**Fig 7:**
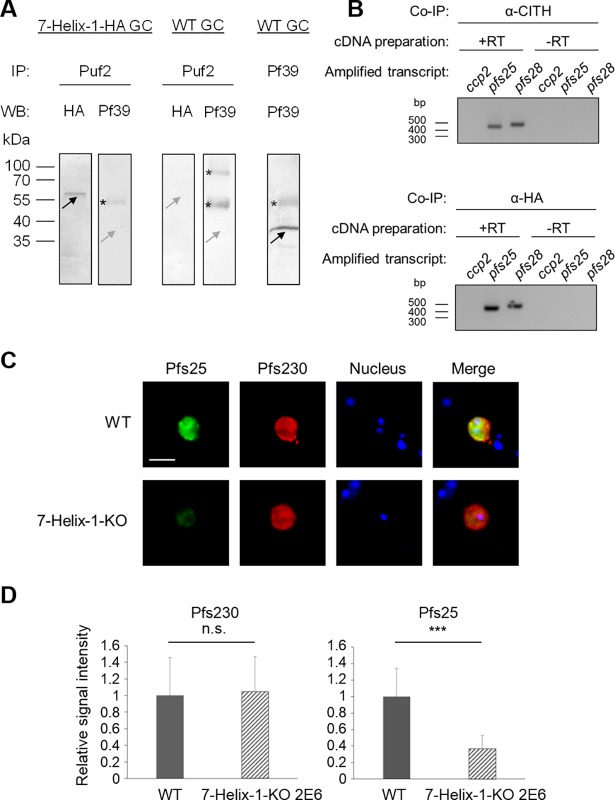
Complex formation of 7-Helix-1 with Puf2 and repressed *pfs25* mRNA and Pfs25 synthesis. (A) Co-immunoprecipitation of 7-Helix-1 with Puf2. Co-immunoprecipitation assays on lysates of 7-Helix-1-HA or WT NF54 gametocytes were employed using either polyclonal mouse anti-Puf2 or anti-Pf39 antisera, followed by WB using rabbit anti-HA antibodies or mouse anti-Pf39 antibody to detect precipitated proteins. Black arrows indicate precipitated proteins, grey arrows indicate expected running lines in the negative controls. Asterisks indicate bands corresponding to the precipitation antibodies. Results are representative of three independent experiments. (B) Association of repressed transcript with 7-Helix-1. RIP assays on lysates of 7-Helix-1-HA gametocytes were employed using either rabbit anti-CITH or rabbit anti-HA antibodies, followed by RNA isolation. RT-PCR was performed amplifying co-eluted transcripts of *ccp2* (198 bp), *pfs25* (459 bp) and *pfs28* (494 bp). cDNA preparation lacking the reverse transcriptase (-RT) was used to prove absence of gDNA contamination. (C, D) Quantitative expression analysis of gametocyte-specific adhesion proteins. (C) Expression of Pfs25 and Pfs230 in activated gametocytes. WT NF54 and 7-Helix-1-KO 2E6 gametocytes at 30 min p.a. were immunolabeled with rabbit anti-Pfs25 (green) and mouse anti-Pfs230 antibodies (red). Nuclei were highlighted by Hoechst33342 nuclear stain (blue). Bar, 5 μm. (D) Quantitative IFA analysis of Pfs25 and Pfs230. IFAs were employed as described above and the fluorescence intensities for Pfs25 and Pfs230 of 20 immunolabeled activated gametocytes were measured in triplicate using ImageJ (mean ± SD). The respective signal in WT lysates was set to 1. *** p ≤ 0.001 (Student‘s t-test). Results (in A-C) are representative of two to three independent experiments.

The Puf2 antibody was then employed in co-immunoprecipitation assays, using the 7-Helix-1-HA line. Subsequent WB demonstrated the presence of HA-tagged 7-Helix-1 in the precipitates, using the anti-HA antibody, while no Pf39 signal was detected, when the anti-Pf39 antisera was used for control (Fig 7A). Additional controls included Puf2-antibody-based precipitation of WT gametocyte lysate, which did not result in any co-precipitation of 7-Helix-1-HA or Pf39 as well as the successful precipitation of Pf39 in WT gametocyte lysates using the corresponding antibody (Fig 7A).

We then analyzed, if *pfs25* transcript is also associated with 7-Helix-1. RNA-co-immunoprecipitation (RIP) assays followed by RT-PCR were employed, using the 7-Helix-1-HA line. When antibodies directed against the HA-tag or against CITH for positive control were used in the RIP assays and cDNA was prepared from co-precipitated transcript, subsequent RT-PCRs amplified *pfs25* transcript (Fig 7B). Furthermore, transcript for *pfs28*, encoding the Pfs25 paralog Pfs28 also known to be repressed in translation during gametocyte maturation [51], was amplified. However, no *pfccp2* transcript encoding the gametocyte-specific protein PfCCp2 was detected. Preparations lacking reverse transcriptase were used for negative control in the assays, and no PCR products were detected (Fig 7B).

We then explored, if the interaction between 7-Helix-1 and Puf2 affects Pfs25 synthesis. IFAs were employed and demonstrated a reduced immunolabelling for Pfs25 in 7-Helix-1-KO gametocytes at 30 min p.a. compared to WT (Fig 7C). No reduction in immunolabeling, however, was observed, when antisera against the gametocyte-specific protein Pfs230 was used. The fluorescence intensities for Pfs25 and Pfs230 were measured by Image J1.51f in three independent experiments in activated gametocytes at 30 min p.a. of WT and the 7-Helix-1-KO line 2E6. The quantifications revealed significantly lower Pfs25 levels in gametocytes lacking 7-Helix-1 compared to WT, while no difference in the Pfs230 levels in the same gametocytes was detected (Fig 7D). We thus conclude that the interaction of 7-Helix-1 and Puf2 affects Pfs25 synthesis.

## Discussion

The human-to-mosquito transmission of *P*. *falciparum* requires a high degree of tight coordination allowing the parasite to rapidly adapt to the changing environment. The parasite prepares for transmission by the formation of intraerythrocytic gametocytes, which immediately convert into male and female gametes, once taken up by the blood-feeding mosquito. In order to pre-adapt to the change of host, female gametocytes store several hundred mRNAs in specific SGs via translational repressors like DOZI, CITH or Puf2. These repressed mRNAs code for proteins required for further development in the mosquito, particularly the ookinete and oocyst stages ([14–16]; reviewed in [12]). With the onset of gametogenesis, the repressed mRNAs become accessible again for ribosomes, hence female gametogenesis requires a rapid increase in translational activities to ensure parasite survival. Despite the crucial function of the gametocyte-specific SGs for parasite transmission and thus spread of the tropical disease, to date not much is known about their compositions and the molecules involved in the translational fine control.

Here we identified the heptahelical protein 7-Helix-1 of *P*. *falciparum* as a novel SG component of female gametocytes. As an integral part of these SGs, 7-Helix-1 interacts with translational repressors, like CITH, PABP1, DOZI and Puf2, and is thereby involved in the regulation of translational processes during sexual reproduction. Consequently, gametocytes deficient of 7-Helix-1 are severely impaired in female gametogenesis and thus transmission to the mosquito. Further, 7-Helix-1-KO gametocytes exhibit a transcriptional deregulation of proteins required for translational control. This transcriptional deregulation is particularly evident for ribosomal proteins, but can further be observed for initiation and elongation factors, heat shock proteins, RNA-binding proteins, as well as RNA helicases, all of which represent typical components of SGs in other organisms [52]. Noteworthy in this context, the orthologues of 18 of the transcripts up-regulated in the 7-Helix-1-KO are components of previously identified gametocyte repressome of *P*. *berghei* [53] providing a further link between 7-Helix-1 and gametocyte SGs. In addition, non-coding RNAs, like snoRNAs and tRNAs required for ribosomal assembly and functionality, are down-regulated in their transcription levels. Such a deregulation has previously been observed for tRNAs in other organisms and can either be explained by cellular stress [54] or by feedback regulation due to the high numbers of uncharged tRNAs in the cytosol (reviewed in [55–57]).

In silico analyses demonstrated that 7-Helix-1 is homologous to hLanCL2, a heptahelical regulator of glycemic control. Previous studies reported that hLanCL2 does not represent a transmembrane GPCR, but is located in the cytoplasm of a variety of human cells, including liver, immune and muscle cells. Here, hLanCL2 acts as a receptor for the stress hormone abscisic acid (ABA), which in humans regulates both inflammation and glycemia. Binding of ABA by hLanCL2 leads to a G-protein-mediated increase in cytosolic cAMP and calcium [5,32,58–60]. Further hLanCL2 acts as a positive regulator within the Akt/mTORC2 pathway by enabling the insulin-dependent phosphorylation of Akt via an interaction with the mTORC2-complex, thereby contributing to cell survival [61]. Due to the role of ABA in inflammatory diseases and diabetes, hLanCL2 is currently considered a promising therapeutic target [62–64].

Future studies are required to shed light on the molecular function of 7-Helix-1 during protein synthesis control in female gametocytes and to unveil potential activator and effector proteins of this heptahelical stress regulator. Initial clues might be gained from its homology with hLanCL2. While apicomplexan parasites have lost most of the components of the TORC pathway, including the TOR kinase, through genomic reduction [65,66], a recent study in *P*. *falciparum* described the RNA polymerase III repressor Maf1 as a plasmodial mTORC1 component. Maf1 regulates transcription of this polymerase under stress conditions and parasites deficient in Maf1 are defective in downregulation of tRNA synthesis [67]. Further, the plasmodial Akt kinase PKB is linked to a Ca^2+^/calmodulin-dependent signaling cascade relevant for intraerythrocytic survival of the malaria parasite [68–71], providing the presence of further conserved interaction partners of hLanCL2 in *Plasmodium*.

In conclusion, our combined data demonstrate that 7-Helix-1 constitutes SGs with key functions in translational control during female gametogenesis. Due to its crucial role for human-to-mosquito transmission 7-Helix-1 might represent a novel gametocytocidal drug-target.

## Materials and methods

### Ethics statement

Experiments for the generation of antisera in mice were performed according to the German Animal Welfare Act (Tierschutzgesetz) and were approved by the animal welfare committees of the government of the District Council of Cologne, Germany (ref. no. 84–02.05.30.12.097 TVA). The University Hospital Aachen Ethics commission approved all work with human blood, the donors remained anonymous and serum samples were pooled. Human erythrocyte concentrate and serum used in this study were purchased from the Department of Transfusion Medicine (University Hospital Aachen, Germany).

### Gene identifiers

The following PlasmoDB gene identifiers are assigned to the *P*. *falciparum* genes and proteins analyzed in this study: AMA1 [PlasmoDB: PF3D7_1133400]; PfCCp2 [PlasmoDB: PF3D7_1455800]; Pfs230 [PlasmoDB: PF3D7_0209000]; EXP1 [PlasmoDB: PF3D7_1121600]; MSP1 [PlasmoDB: PF3D7_0930300]; Pf39 [PlasmoDB: PF3D7_1108600]; 7-Helix-1 [PlasmoDB: PF3D7_0525400]; PfAldolase [PlasmoDB: PF3D7_1444800]; Pfs16 [PlasmoDB: PF3D7_0406200]; Pfs25 [PlasmoDB: PF3D7_1031000]; Pfs28 [PlasmoDB: PF3D7_1030900]; FNPA [PlasmoDB: PF3D7_1451600]; Pfg377 [PlasmoDB: PF3D7_1250100]; RNF1 [PlasmoDB: PF3D7_0314700]; HSP70-1 [PlasmoDB: PF3D7_0831700]; M1-AP [PlasmoDB: PF3D7_1311800]; Falcilysin [PlasmoDB: PF3D7_1360800]; CITH [PlasmoDB: PF3D7_1474900]; DOZI [PlasmoDB: PF3D7_0320800]; PABP1 [PlasmoDB: PF3D7_1224300]; Puf2 [PlasmoDB: PF3D7_0417100]; SR1 [PlasmoDB: PF3D7_1131100]; SR10 [PlasmoDB: PF3D7_1215900]; SR12 [PlasmoDB: PF3D7_0422800]; SR25 [PlasmoDB: PF3D7_0713400].

### Bioinformatics

Prediction of the transmembrane domains and intra-/extracellular regions of 7-Helix-1 was performed using the TMHMM Server v. 2.0 (http://www.cbs.dtu.dk/services/TMHMM/); further domain prediction was performed using the Simple Modular Architecture Research Tool (SMART, http://smart.embl.de/; [72]). The 3D modelling of 7-Helix-1 was conducted using the I-TASSER server (http://zhanglab.ccmb.med.umich.edu/I-TASSER/; [73]). For sequence alignment of homologous proteins and the generation of the phylogenetic tree the software MainWorkbench 7.5 was used. Predictions of gene expression and function were made using the database PlasmoDB (http://plasmoDB.org;[74]).

### Antibodies

In this study, the following antisera were used: rabbit polyclonal antisera against MSP-1 (ATCC), EXP1 [75], *Pf*s230 (Biogenes), *Pf*s25 (ATCC), *Pf*s28 (ATCC), *Pf*HSP70-1 [49], *Py*PABP1 [76], *Pf*CCp2 [77], *Pf*g377 ([78], kindly provided by Pietro Alano, Istituto Superiore di Sanità, Rome, Italy), and *Hs*DDX6/*Pf*DOZI (kindly provided by Joseph Reese, Penn State University); mouse polyclonal antisera against *Pf*M1-AP [79], *Pf*s230 [80], *Pf*Falcilysin [79], *Pf*s16 [81]; kindly provided by Pietro Alano, Istituto Superiore di Sanità, Rome, Italy) and *Pf*39 [77]; rabbit anti-GFP antibody (Santa Cruz Biotechnology) and rabbit anti-HA antibody (Sigma-Aldrich). The generation of antisera against 7-Helix-1 (anti-7-Helix-1rp1 and 2), CITH and Puf2 is described below.

### Parasite culture

*P*. *falciparum* strain NF54 (WT NF54) was used in this study. Asexual blood stage parasites and gametocytes of WT NF54, the two 7-Helix-1-KO lines 2E6 and 1D12, the complementation line 7-Helix-1-KO(+) and the 7-Helix-1-HA line (for the generation of the 7-Helix-1 mutant lines, see below) were cultivated *in vitro* in human blood group A+ erythrocytes as previously described [82,83]. All parasite stages were maintained in RPMI1640/HEPES medium (Gibco) supplemented with 10% v/v heat inactivated human A+ serum, 50 μg/ml hypoxanthine (Sigma-Aldrich) and 10 μg/ml gentamicin (Gibco). For cultivation of 7-Helix-1-KO parasites, the selection drug blasticidin (InvivoGen) was added in a final concentration of 5.4 μM; for cultivation of 7-Helix-1-KO(+) and 7-Helix-1-HA parasites, the selection drug WR99210 (Jacobus Pharmaceutical Company) was added in a final concentration of 2.5 nM. All cultures were kept at 37°C in an atmosphere of 5% O_2_ and 5% CO_2_ in N_2_. Gametocytes were enriched via Percoll gradient centrifugation (GE Healthcare Life Sciences) as described previously [84]. Gametogenesis was induced by adding xanthurenic acid in a final concentration of 100 μM dissolved in 1% v/v 0.5 M NH_4_OH/ddH_2_O and incubation for 15 min at room temperature (RT).

### Generation of the 7-Helix-1 mutant lines

**7-Helix-1-KO:** 7-Helix-1-KO parasites were generated via single cross-over homologous recombination using the pCAM-BSD vector [85–88]. A 534-bp gene fragment homologous to a region of the N-terminus of the gene was amplified via PCR using the respective 7-Helix-1-KO primers (for primer sequences, see S2 Table). Ligation of insert and vector backbone was mediated by BamHI and NotI restriction sites (underlined). A NF54 WT culture synchronized for 5% ring stages was loaded with 60 μg of the vector in transfection buffer via electroporation (310 V, 950 μF, 12 ms; Bio-Rad gene-pulser) as described [87]. Starting at 6 h post-transfection, blasticidin (Invivo-Gen) was added to a final concentration of 5.4 μM. A mock control was electroporated using transfection buffer without the disruption vector and cultured in medium without selection drug. After approximately 10 weeks, blasticidin-resistant parasites appeared in culture. To check for successful plasmid integration into the *7-helix-1* gene locus, genomic DNA (gDNA) of transfected parasites was isolated using the NucleoSpin Blood Kit (Macherey-Nagel) following the manufacturer’s protocol and used as template in diagnostic PCR. The following primers were used to confirm vector integration: 7-Helix-1-KO-5’-integration forward primer (1), 7-Helix-1-KO-3’-integration reverse primer (2), pCAM-BSD forward primer (3) and pCAM-BSD reverse primer (4) (for primer sequences, see S2 Table). After confirmation of plasmid integration, a mixed culture with >3% ring stages was diluted and transferred to a 96-well-plate. After three weeks of cultivation, clones were identified via Malstat assay (see below) and subsequent diagnostic PCR was performed to confirm vector integration and the absence of the WT *7-helix-1* gene locus. Two clonal lines, 7-Helix-1-KO 1D12 and 2E6, were isolated.

#### 7-Helix-1-KO(+)

The generation of the 7-Helix-1-KO(+) complementation line was performed by transfecting the 7-Helix-1-KO clone 1D12 with an episomal copy of the *7-helix-1* gene (pARL2-FNPA5’-7-Helix-1-GFP). Therefore, the promotor region of the gametocyte-specific protein FNPA [34] and the *7-helix-1* coding region were ligated into the backbone of the pARL2-GFP vector [89,90] to receive vector pARL2-FNPA5’-GFP. For the amplification of the *fnpa* 5’-promotor region, the FNPA5’ forward and reverse primers were used (for primer sequences, see S2 Table). Ligation with the vector was mediated by NotI and XhoI restriction sites (underlined). For the amplification of *7-helix-1*, 7-Helix-1-KO(+) forward and reverse primers were used (for primer sequences, see S2 Table). Ligation with the vector was mediated by XhoI and AvrII restriction sites (underlined). Transfection was performed as described above. Diagnostic PCR to confirm uptake of the pARL2-FNPA5’-7-Helix-1-GFP plasmid in the complementation line was performed on DNA isolated from mixed asexual cultures. The plasmid was detected (831 bp) using the 7-Helix-1-GFP forward and reverse primers. Amplification of *pfama1* (189 bp) with the above mentioned primers was used as positive control (for primer sequences, see S2 Table).

#### 7-Helix-1-HA

C-terminal 3xHA-Streptavidin-tagging of 7-Helix-1 was performed via single cross-over homologous recombination using the pHAST vector [91]. Therefore, a 588-bp gene fragment homologous to the C-terminus of the gene lacking the stop codon was amplified using 7-Helix-1-HA forward and reverse primers (for primer sequences, see S2 Table). Ligation with the vector was mediated by SacII and XhoI restriction sites (underlined). Transfection was performed as described above and successful integration of the vector was confirmed by integration PCR using 7-Helix-1-HA-5’-integration forward primer (1), 7-Helix-1-HA-3’-integration reverse primer (2), pHAST forward primer (3) and pHAST reverse primer (4) (for primer sequences, see S2 Table).

### Mosquito maintenance and standard membrane feeding assay

*Anopheles stephensi* mosquitoes were maintained as described previously [87]. Briefly, rearing of the mosquitoes was conducted under standard insectary conditions at 26 ± 0.5°C, 80 ± 2% humidity and a 12/12 h light/dark cycle. Egg production was induced by feeding adult female mosquitoes with non-infected human erythrocyte cultures and eggs were collected on filter paper in a beaker containing 0.1% w/v sea salt solution four days after the blood meal. Emerged larvae were reared with a density of 300 larvae/tray (3 l) in 0.1% w/v sea salt solution and fed on fodder pellets. After transformation, pupae were collected and placed in cages for mosquito emergence. Feeding of adult mosquitoes was performed using a cotton pad soaked with 5% w/v sterile saccharose solution supplemented with para-aminobenzoic acid and 40 μg/ml gentamicin [92]. For SMFAs, mature gametocytes of WT NF54 and the 7-Helix-1-KO line 2E6, which were positively tested for exflagellation activity, were enriched and the parasitemia was adjusted to 0.25% by the addition of human erythrocytes in A+ serum. The mosquitoes were allowed to feed on the cell suspension via glass feeders for 20 min [93]. For ookinete quantification, samples of the midgut were prepared at 24 h p.i. and subjected to IFA. Ookinetes were immunolabeled with rabbit anti-Pfs28 antisera and counted in 30 optical fields for four times. To determine the numbers of oocysts, midguts were dissected at day 10 p.i. and stained with 0.2% v/v mercurochrome/PBS. For sporozoite quantification, salivary glands were prepared at day 17 p.f. and parasites were counted using a Neubauer chamber.

### Recombinant protein expression

Two recombinant 7-Helix-1 proteins, spanning amino acids 234–320 (7-Helix-1rp1) and 1–467 (7-Helix-1rp2) were expressed as fusion proteins with an N-terminal maltose binding protein (MBP)-tag using the pIH902 vector [80] for 7-Helix-1rp1 or with an N-terminal glutathione-S-transferase (GST)-tag using the pGEX-4T-1 vector (Amersham Bioscience) for 7-Helix-1rp2. Gene fragments were amplified from *P*. *falciparum* gDNA using the respective primers, resulting in gene fragments of 279 bp and 1426 bp, respectively. Ligation into the expression vectors was mediated by BamHI/PstI restriction sites (underlined) for 7-Helix-1rp1 into pIH902 and by BamHI/XhoI restriction sites (underlined) for 7-Helix-1rp2 into pGEX-4T-1. Recombinant Puf2rp1 protein (amino acids 243–424) was expressed as fusion protein with an N-terminal MBP-tag using the pMAL^TM^c5X vector (New England Biolabs). Amplification of the gene fragment from *P*. *falciparum* gDNA resulted in a 579 bp gene fragment. Ligation into the pMAL^TM^c5X expression vector was mediated by XmnI/PstI restriction sites (underlined). Expression of the recombinant fusion proteins was performed in *Escherichia coli* BL21 (DE3) RIL cells (Stratagene) according to the manufacturer’s protocol. The fusion proteins were purified via affinity chromatography from bacterial extracts using amylose resin (New England Biolabs) for 7-Helix-1rp1 and Puf2, and glutathione-sepharose (GE Healthcare) for 7-Helix-1rp2 according to the manufacturer’s protocols. Successful purification of the proteins was validated by SDS-PAGE. Recombinant *P*. *yoelii* CITH protein (amino acid 1–100) was expressed and rabbit antibody was generated using a method described in [76]. Primer sequences used for the expression of recombinant proteins are listed in S2 Table.

### Generation of antisera

Recombinant fusion proteins 7-Helix-1-rp1-MBP, 7-Helix-1-rp2-GST and Puf2-rp1-MBP were purified via affinity chromatography as described above followed by PBS buffer exchange via filter centrifugation using Amicon Ultra 15 (Sigma-Aldrich) according to the manufacturer’s protocol. Six weeks-old female NMRI mice (Charles River Laboratories) were immunized with subcutaneous injections of 100 μg recombinant protein emulsified in Freund’s incomplete adjuvant (Sigma-Aldrich) followed by a boost after 4 weeks with 50 μg recombinant protein. At day 10 after the boost, mice were anesthetized by intraperitoneal injection of a mixture of ketamine and xylazine according to the manufacturer’s protocol (Sigma-Aldrich) followed by the collection of polyclonal immune sera via heart puncture. The immune sera of three mice immunized with the same antigen were pooled; NMS were used as a negative control.

### Co-immunoprecipitation assay

Co-immunoprecipitation assays were performed to precipitate either protein or RNA. Gametocyte lysates of WT NF54 and the 7-Helix-1-HA line were obtained as described above. For the precipitation of proteins, the lysates were initially incubated with 5% v/v pre-immune mouse or rabbit sera and 20 μl of protein G-beads (Roche) for 1 h at 4°C. After centrifugation, the supernatant was incubated for 1 h at 4°C with 5% v/v polyclonal mouse antisera against 7-Helix-1, Puf2 as well as Pf39 [94] used as a negative control, or polyclonal rabbit antisera against CITH, DOZI or HA. A volume of 20 μl protein G-beads was added and incubated overnight at 4°C. The beads were centrifuged, washed with PBS five times and resuspended in an equal volume of loading buffer. The sample was then subjected to WB (see below). For the precipitation of RNA (RIP; [53]), the beads were resuspended in Trizol instead of loading buffer, and RNA isolation and diagnostic RT-PCR were performed as described below.

### Diagnostic RT-PCR

Either asexual blood stages of synchronized WT NF54 cultures were harvested, immature and mature gametocytes were enriched via Percoll gradient purification, and activated gametocytes were collected at 15 min p.a. Total RNA was isolated using the Trizol reagent (Invitrogen) according to the manufacturer’s protocol or the co-precipitated RNAs from the RIP assays were used [53]. To remove gDNA contamination, samples were treated with RNase-free DNase I (Qiagen), followed by phenol/chloroform extraction and ethanol precipitation. Photometric analysis revealed A260/280 ratios greater than 2.1. The cDNA synthesis was performed with two micrograms of each RNA sample using the SuperScript III First-Strand Synthesis System (Invitrogen) following the manufacturer’s protocol. Transcript for *7-helix-1* (527 bp), *pfs25* (459 bp) and *pfs28* (494 bp) was amplified in 25–35 cycles using the respective primers (for primer sequences, see S2 Table). To confirm purity of the asexual blood stage and gametocyte samples, transcript amplification of *pfama1* (189 bp) [33] and of *pfccp2* (198 bp) [34] using the respective primers were performed (for primer sequences, see S2 Table). Amplification of *pfaldolase* (378 bp) was used as loading control and to investigate potential contamination with gDNA in the negative control lacking reverse transcriptase (S16A Fig). PCR products were separated by 1.2% w/v agarose gel electrophoresis.

### Real-time RT-PCR

Total RNA was isolated from Percoll-purified, mature gametocytes of WT NF54 and the 7-Helix-1-KO line 2E6 as described above. The gametocytes were either non-activated or harvested at 30 min p.a. One μg of each total RNA sample was used for cDNA synthesis using either the SuperScript III or SuperScript IV First-Strand Synthesis System (Invitrogen), following the manufacturer's instructions. The synthesized cDNA was first tested by diagnostic RT-PCR for asexual blood stage contamination using primers specific for the gene encoding the apical membrane antigen AMA-1 [33] and for gametocyte specificity using primers specific for the gene encoding the LCCL-domain protein PfCCp2 [34] (S16B Fig). Controls without reverse transcriptase were used to investigate potential gDNA contamination by using *pfaldolase* primers (for primer sequences, see S2 Table). Primers for quantitative real time RT-PCR were designed using the Primer 3 software (http://frodo.wi.mit.edu/primer3/) and tested on gDNA in conventional PCR to confirm primer specificity (for primer sequences, see S2 Table). Real time RT-PCR measurements were performed using either the Bio-Rad iQ5 Real-Time Detection System or the StepOnePlus Real-Time PCR System (Applied Biosystems). Reactions were performed in a total volume of 20 μl using the maxima SYBR green qPCR master mix according to manufacturer’s instructions (Thermo Scientific) in triplicate. Controls without template and without reverse transcriptase were included in all real time RT-PCR experiments. Transcript levels were calculated by the 2^-ΔCt^ method [95], the Ct was normalized with the Ct of the gene encoding seryl tRNA-ligase (PF3D7_0717700) as reference [96,97] and fold changes were calculated via the normalized Ct ratio of 7-Helix-1-KO:WT.

### Indirect immunofluorescence assay

Mixed asexual blood stage and gametocyte stage cultures as well as macrogametes and zygotes of WT NF54, the 7-Helix-1-KO lines 2E6 and 1D12 and the 7-Helix-1-KO(+) line collected at 2, 5, 10, 30 min (macrogametes) and 4 h (zygotes) p.a., and ookinetes obtained from mosquito midguts at 24 h p.i. were subjected to IFAs. After the cell monolayers were air-dried on glass slides, they were fixed with 4% w/v paraformaldehyde/PBS (pH 7.4) for 10 min at RT followed by membrane permeabilization with 0.1% v/v Triton X-100/125 mM glycine (Carl Roth)/PBS at RT for 30 min. Blocking of non-specific binding sites was performed using 3% w/v BSA/PBS for 1 h, followed by incubation with polyclonal mouse antisera against 7-Helix-1rp1 (dilution 1:50), 7-Helix-1rp2 (dilution 1:20), Puf2rp1 (dilution 1:20), Pfs230 (dilution 1:200), with polyclonal rabbit antisera against the HA-tag (dilution 1:50) or with NMS (dilution 1:20) for 2 h at 37°C. Binding of primary antibody was detected by incubating the samples with monoclonal Alexa Fluor 488-conjugated goat anti-mouse or anti-rabbit IgG antibodies (dilution 1:1,000; Molecular Probes) for 1 h at RT. The different parasite stages were detected by double-labelling with stage-specific polyclonal rabbit or mouse antisera, i.e. anti-MSP1 antisera (dilution 1:1,000), anti-EXP1 antisera (dilution 1:100), anti-Pfs230 antisera (dilution 1:200), anti-Pfs25 antisera (dilution 1:1,000), anti-Pfs28 antisera (dilution 1:200), anti-CITH antisera (dilution 1:1,000), anti-PABP1 antisera (dilution 1:1,000), anti-DOZI antisera (1:500) and anti-Pfg377 antisera (1:500), followed by incubation with monoclonal Alexa Fluor 594-conjugated goat anti-rabbit or anti-mouse IgG antibodies (dilution 1:1,000; Molecular Probes). Nuclei were highlighted by treatment with Hoechst33342 nuclear stain for 1 min at RT and cells were mounted with anti-fading solution AF2 (Citifluor Ltd) and sealed with nail varnish. For sex-specific quantifications, 100 gametocytes labelling for either Pfs230 or Pfs25 were counted in ten replicates and the percentage of 7-Helix-1-positive cells was calculated. Data analysis was performed using MS Excel 2013. IFAs were analyzed using a Leica DM 5500B fluorescence microscope and digital images were processed using Adobe Photoshop CS5 software.

### Subcellular fractioning

For subcellular fractioning, ~ 3 x 10^7^ gametocytes of the 7-Helix-1-HA line were purified as described above and liberated from the RBC in 0.03% saponin/PBS for 3 min at 37°C. Cells were pelleted and lysed by resuspension in 100 μl of 5 mM Tris-HCl (pH 8) supplemented with protease inhibitor cocktail (complete EDTA-free, Roche) and 10 min incubation at RT followed by freezing and thawing. Soluble proteins were separated by centrifugation and the pellet was resuspended in 100 μl of 1% Triton X-100 and incubated for 30 min at RT to extract integral proteins, and the final pellet representing the insoluble proteins was resuspended in 100 μl of 0.5 x PBS/4% SDS/ 0.5% Triton X-100. The samples were then subjected to WB (see below).

### Stress granule core fraction enrichment

SG core fraction enrichment was performed following a previously published protocol [98]. Gametocytes of line 7-Helix-1-HA were purified via Percoll gradient as described above and stress was induced by sodium arsenite treatment (0.5 mM) for 1 h at 37°C. Cells were pelleted and washed with RPMI1640. The cell pellet was flash-frozen in liquid N_2_ and stored at -80°C if necessary. For the enrichment of the SG core fraction, the cell pellet was thawed on ice, resuspended in 100 μl SG lysis buffer (50 mM Tris HCl pH 7.4, 100 mM potassium acetate, 2 mM magnesium acetate, 0.5 mM DTT, 50 μg/ml heparin, 0.5% NP40, 1 complete mini EDTA free protease inhibitor tablet per 50 ml of lysis buffer (Roche), RNasin 1 U/μl (Promega)) and lysed by five passages through a 26G needle. Cell debris was pelleted by centrifugation at 1000g for 5 min at 4°C and the supernatant was transferred to a new microcentrifuge tube. After centrifugation at 18,000g for 20 min at 4°C, the pellet was resuspended in 100 μl SG lysis buffer followed by another centrifugation step at 18,000g for 20 min at 4°C. The pellet was resuspended in 100 μl SG lysis buffer and centrifuged at 850g for 2 min at 4°C. The supernatant represented the SG core enriched fraction and was subjected to WB (see below).

### Western blot analysis

Asexual blood stage parasites of WT NF54, the 7-Helix-1-KO lines 2E6 and 1D12, the complementation line 7-Helix-1-KO(+) and the 7-Helix-1-HA line were harvested from mixed or synchronized cultures, while gametocyte stages were enriched by Percoll purification. Parasites were incubated with 0.05% w/v saponin/PBS for 10 min for erythrocyte lysis. Pelleted parasites were resuspended in lysis buffer (150 mM NaCl, 0.1% v/v Triton X-100, 0.5% w/v sodium deoxycholate, 0.1% w/v SDS, 50 mM Tris-HCl pH 8.0) supplemented with protease inhibitor cocktail (complete EDTA-free, Roche) and incubated for 10 min on ice. Lysed non-infected erythrocytes were used as negative control. 5x SDS-PAGE loading buffer containing 25 mM dithiothreitol was then added to the lysates, heat-denatured for 10 min at 95°C, and separated via SDS-PAGE and transferred to Hybond ECL nitrocellulose membrane (Amersham Biosciences) according to the manufacturer’s protocol. Blocking of non-specific binding sites was performed by incubation with 5% w/v skim milk and 1% w/v BSA in Tris-buffered saline (pH 7.5) overnight at 4°C. For immunodetection, membranes were incubated for 2 h at RT with polyclonal mouse anti-7-Helix-1rp2 antisera (dilution 1:1,000), polyclonal mouse anti-Pf39 antisera (dilution 1:500), polyclonal mouse anti-PfM1-AP antisera (dilution 1:50), polyclonal mouse anti-Falcilysin antisera (dilution 1:50), polyclonal mouse anti-Pfs16 antisera (dilution 1:100), rabbit anti-HSP70-1 antibody (dilution 1:5,000), rabbit anti-HA antibody (dilution 1:200), rabbit anti-CITH antibody (dilution 1:1,000), rabbit anti-PABP1 antibody (dilution 1:1,000), rabbit anti-CCp2 antisera (1:1,000) or rabbit anti-GFP antibody (dilution 1:1,000) in blocking solution or TBS/0.1% Tween-20. Following several washing steps, the membranes were incubated for 1 h at RT with a goat anti-mouse or anti-rabbit alkaline phosphatase-conjugated secondary antibody (dilution 1:10,000; Sigma-Aldrich) and developed in a solution of nitroblue tetrazolium chloride (NBT) and 5-bromo-4-chloro-3-indoxyl phosphate (BCIP; Roche) for 15 min at RT. Blots were scanned and processed using Adobe Photoshop CS5 software. For quantification of band intensities ImageJ 1.51f was used; data analysis was performed using MS Excel 2013.

### Malstat assay

To determine the antimalarial effect and inhibitory concentrations of the translation inhibitors emetine and cycloheximide (Sigma-Aldrich), a Malstat assay was performed as described previously [99]. *P*. *falciparum* WT NF54 cultures synchronized for ring stages were plated in triplicate in 96-well plates (200 μl/well) at a parasitemia of 1% in the presence of inhibitors in concentrations ranging from 500 μM to 0.5 nM. Chloroquine, dissolved in double-distilled water, served as a positive control in the experiments; incubation of parasites with the solvents alone (double-distilled water for emetine, ethanol for cycloheximide) was used as a negative control. After cultivation for 72 h, the parasites were resuspended, and aliquots of 20 μl were removed and added to 100 μl of the Malstat reagent in a 96-well microtiter plate. Parasite lactate dehydrogenase activity was evaluated by adding 20 μl of a mixture of NBT (nitroblue tetrazolium) and diaphorase (1:1; 1 mg/ml stock each) to the Malstat reaction, and measurement of optical densities at 630 nm. Each compound was tested three times, and the IC_50_ values were calculated from variable-slope sigmoidal dose-response curves using the GraphPad Prism 5 program.

### Asexual blood stage replication and gametocyte development assay

For the comparison of asexual blood stage replication and the development of gametocytes between WT NF54 and the 7-Helix-1-KO lines 1D12 and 2E6, blood stage cultures were synchronized and set to an initial parasitemia of 2% ring stages. To compare asexual blood stage replication, Giemsa-stained thin blood smears were prepared over a time span of 49 h at seven different points (0, 15, 20, 25, 39, 44, and 49 h post-seeding); to compare the development of gametocytes, Giemsa-stained thin blood smears were prepared over a time span of 15 days at six different time points (6, 7, 8, 10, 13, and 15 d post-seeding). The smears were analyzed by light-microscopy at 1,000-fold magnification and 50 parasites were counted four times and grouped according to their developmental stage.

### Comparative exflagellation assay

Mature gametocytes of the WT NF54 and the 7-Helix-1-KO line 2E6 were activated *in vitro* with 100 μM xanthurenic acid. At 15 min p.a. the numbers of exflagellation centers were examined microscopically and counted at 400-fold magnification in 30 optical fields for four times. The relative numbers of exflagellation centers were calculated (WT set to 100%). Four independent experiments were conducted; data analysis was performed using MS Excel 2013.

### Macrogamete and zygote formation assay

For the analysis of macrogamete and zygote formation, mature gametocytes of WT NF54, the 7-Helix-1-KO lines 2E6 and 1D12 and the complementation line 7-Helix-1-KO(+) were enriched via Percoll purification and set to a gametocytemia of 2–4%. For inhibitor experiments, the parasites were incubated with emetine or cycloheximide at IC_50_ concentration (as determined by Malstat assay) for 30 min at 37°C. Incubation with the solvents alone was used as a negative control. The gametocytes were activated and at 30 min p.a. (macrogametes) or 4 h p.a. (zygotes), the samples were subjected to IFA. Immunolabeling was performed as described above and macrogametes and zygotes were labelled with rabbit anti-Pfs25 antisera. Parasites were counted microscopically using a Leica DM 5500B fluorescence microscope with 600-fold magnification. For comparative macrogamete and zygote formation assays, 30 optical fields were counted for three to four times. The relative numbers of macrogametes and zygotes were calculated (WT set to 100%). Three to eight independent experiments were conducted; data analysis was performed using MS Excel 2013 and GraphPad Prism 5. For inhibitor experiments, macrogametes and zygotes per 1,000 RBC were counted for five times. Two independent experiments were performed; data analysis was performed using MS Excel 2013 and GraphPad Prism 5.

### Transmission electron microscopy

Mature gametocytes of NF54 WT and the 7-Helix-1-KO line 2E6 were enriched via Percoll purification and activated as described above. Samples were collected at 0 and 30 min p.a. and fixed with 1% v/v glutaraldehyde and 4% w/v paraformaldehyde/PBS (pH 7.4) overnight at 4°C. Post-fixation of the specimens was performed with 1% v/v osmium tetroxide and 1.5% w/v K_3_Fe(CN)_6_ in PBS for 2 h at RT, followed by incubation in 0.5% w/v uranyl acetate for 1 h. For dehydration of the specimens, increasing concentrations of ethanol (70%/80%/95%/100%) were used, followed by an incubation step for 1 h in propylene oxide and another 1 h incubation step in a 1:1 mixture of propylene oxide and Epon (Sigma-Aldrich). Subsequently, specimens were embedded in Epon at 60°C for 48 h. Ultrathin sections were cut with a Leica ultramicrotome Ultracut UCT and post-stained with 1% w/v uranyl acetate for 30 min and 0.2% w/v lead citrate for 15 s. Examination of the sections was performed using a CM100 transmission electron microscope (FEI) and images were recorded digitally with a Quemesa TEM CCD camera and iTEM software (Olympus Soft Imaging Solutions). Alternatively, samples were analysed with a Zeiss EM10 transmission electron microscope and the photographs taken were scanned and processed using Adobe Photoshop CS software.

### Microarray analysis

Mature gametocytes of the WT NF54 and the 7-Helix-1-KO line 2E6 were enriched using Percoll purification and the purity of the gametocyte samples was confirmed via Giemsa smears (S16C Fig). The gametocytes were activated and at 30 min p.a., total RNA was isolated as described above. Quality of RNA samples were assessed using a ND-1000 (NanoDrop Technologies, Thermo Scientific) and by agarose gel electrophoresis. The microarray experiments were carried out as described previously [43,96]. Briefly, first strand amino-allyl cDNA was synthetized using SuperScript II reverse transcriptase (Invitrogen) and then cleaned and concentrated using the Zymo DNA clean and concentrator-5 column (Zymo Research) followed by coupling with Cy5 dye (GE Healthcare). The reference pool consisted of a mixture of RNA from asexual blood stages and gametocytes in which synthesis of first strand amino-allyl cDNA was performed as described above and coupled with Cy3 dye. Equal amounts of Cy5-labelled sample and Cy3-labelled reference pool were subjected to array hybridization for 17 h at 65°C using a *P*. *falciparum* DNA Agilent microarray chip (Agilent Technologies, AMADID #037237) containing the 5,363 coding genes [42]. The Agilent G2600D microarray scanner (Agilent Technologies) was used to scan the arrays. Normalized intensities were extracted using the Agilent feature extractor software version 11.5.1.1 and uploaded to the Princeton University Microarray Database (PUMA.princeton.edu) for analysis. After background subtraction, the log2 of the (Cy5/Cy3) intensity ratio was extracted and the transcript abundance of 7-Helix-1-KO samples was compared to that of WT NF54 samples. For the selection of up- and down-regulated genes, a cut-off value of greater than 1.5-fold applied. Data were analysed using MS Excel 2010. The database PlasmoDB (http://plasmodb.org/plasmo; [74]) was used for gene annotation analyses. GO enrichment analysis for deregulated genes was performed using the PlasmoDB GO analysis tool, p-value < 0.05. Genes with high transcription in gametocytes were defined by transcript levels > 50% of peak transcript levels as published previously [100].

### Fluorescence in situ hybridization assays

The mRNA-FISH-IFA was performed following previously published protocol [101]. WT NF54 or 7-Helix-1-HA gametocytes were liberated from the enveloping RBC by lysis with 0.05% saponin/PBS followed by three washing steps in RPMI1640/HEPES medium (Gibco). Cell monolayers were air-dried on glass slides and fixed with 100% methanol. Permeabilization was performed with 0.1% v/v Triton X-100/125 mM glycine (Carl Roth)/PBS at RT for 10 min. After incubation in 2x SSC for 10 min at RT, hybridization was carried out by incubation of the sample with hybridization buffer (50% deionized formamide/200 μM dextran sulfate (MW = 500,000 g/mol) in 20x SSPE buffer) containing 1 μg biotinylated oligo-dT_25_ probe (Promega) in a humidity chamber overnight at 37°C. The slides were washed twice with 2x SSC for 30 min and once with 0.5x SSC for 15 min at RT, followed by the addition of Alexa Fluor 594-conjugated streptavidin (dilution 1:500; Thermo Scientific) in 4x SSC for 1 h at RT. Washing of the slides was performed twice with 4x SSC for 10 min and once with 4x SSC with 0.01% Triton X-100 for 10 min at RT. Counterlabelling was performed by incubation with mouse anti-7-Helix-1rp2 antisera (dilution 1:50), rabbit anti-Pfs230 antisera (dilution 1:200) or rabbit anti-HA antibody (Sigma-Aldrich, dilution 1:50) in 1% blocking reagent (Roche) in maleic acid buffer for 1 h at 37°C. Following three washing steps in maleic acid buffer, binding of primary antibody was detected by incubating the samples with Alexa Fluor 488-conjugated goat anti-rabbit or anti-mouse IgG antibodies (dilution 1:1,000; Molecular Probes) diluted in 1% blocking reagent (Roche) in maleic acid buffer for 1 h at RT. The samples were washed with maleic acid buffer for three times and nuclei were highlighted by incubation with Hoechst nuclear stain 33342 for 1 min at RT. Subsequently, cells were mounted with anti-fading solution AF2 (Citifluor Ltd) and sealed with nail varnish. Samples were analyzed using a Leica DM 5500B fluorescence microscope and digital images were processed using Adobe Photoshop CS5 software.

### Statistical analysis

Data are expressed as means ± SD. Statistical differences were determined using two-tailed Mann-Whitney-U test, unpaired two-tailed Student’s t-test or One-Way ANOVA with Post-Hoc Bonferroni Multiple Comparison test, as indicated. P values < 0.05 were considered statistically significant. Significances were calculated using GraphPad Prism and are represented in the figures as follows: ns, p > 0.05; * p < 0.05; ** p < 0.01; *** p < 0.001; **** p < 0.0001.

## Supporting information

S1 Fig Sequence alignment of 7-Helix-1. Amino acid sequence alignment of 7-Helix-1 and related proteins from other organisms generated with *CLC MainWorkbench 7*.*5*. The degree of conservation of the single amino acids is shown in color codes (0–30%, red; 30–60%, black; 60–100%, blue). The conserved LanCL domain was identified with the *Simple Modular Architecture Research Tool-SMART* and is highlighted in red. The seven conserved GXXG motifs are highlighted in green. The highly conserved motifs HG in repeat 4 (1), WCXG in repeat 5 (2) and CHG in repeat 6 (3) are labelled with brackets.
(TIF)

S2 Fig *In silico* analysis of 7-Helix-1. (A) Phylogenetic tree of 7-Helix-1. Phylogenetic analysis was performed using *CLC MainWorkbench 7*.*5*. The values on the branching points of the tree are corresponding to the relative consensus during 1000 bootstraps. (B) Modelling of the 3D structure of 7-Helix-1. The 3D structure was generated based on the amino acid sequence and the crystal structure of hLanCL1 using the I-TASSER server. The sequence is shown from the N-terminus (blue) to the C-terminus (red). Left panel, side view; right panel, top view.
(TIF)

S3 Fig Sub-cellular localization of 7-Helix-1 in gametocytes. Gametocytes (GC stage II–V) and activated gametocytes (aGC; at 2, 10 and 15 min p.a.) of WT NF54 were immunolabeled with mouse anti- 7-Helix-1rp2 antisera (green) and counterlabeled with rabbit antibodies directed against Pfs230 and Pfs25 (red). Nuclei were highlighted with Hoechst33342 nuclear stain (blue). Bar, 5 μm. Results are representative of five independent experiments.
(TIF)

S4 Fig Expression analysis of 7-Helix-1 in the *P*. *falciparum* asexual blood stages. Trophozoites (TZ), immature (imSZ) and mature (mSZ) schizonts of WT NF54 were immunolabeled with mouse anti-7-Helix-1rp1 (A) or anti-7-Helix-1rp2 (B) antisera (green) and counterlabeled with rabbit anti-EXP1 antibody (red). Nuclei (in A and B) were highlighted with Hoechst33342 nuclear stain (blue). Bar, 5 μm. Results are representative of five independent experiments.
(TIF)

S5 Fig IFA controls using sera from non-immunized mice. Trophozoites (TZ), schizonts (SZ) and gametocytes of stages II-V (GCII—GCV) of WT NF54 were incubated with NMS (green) and counterlabeled with rabbit antibodies directed against-EXP1 and Pfs230 as indicated (red). Nuclei were highlighted with Hoechst33342 nuclear stain (blue). Bar, 5 μm. Results are representative of five independent experiments.
(TIF)

S6 Fig Generation of the 7-Helix-1-HA line. (A) Schematic depicting the single-crossover homologous recombination strategy for *7-helix-1* fusion to the 3xHA-Streptavidin-tag sequence. The numbered arrows indicate positions of primers used to confirm integration of the 7-Helix-1-HA-pHAST vector. (B) Confirmation of gene locus integration of the 7-Helix-1-HA-pHAST vector. Diagnostic PCR was used to demonstrate vector integration. Primers 1 and 4 were used to demonstrate 5‘-integration and primers 2 and 3 were used to demonstrate 3‘-integration. Primers 3 and 4 were used to detect the episomal plasmid, while primers 1 and 2 were used for amplification of the WT *7-helix-1* locus. The gDNA of a WT NF54 was used as a negative control. (C) Localization of 7-Helix-1 in the 7-Helix-1-HA line. Mature gametocytes of WT NF54 and the 7-Helix-1-HA line were immunolabeled with rabbit anti-HA antibodies (green) and counterlabeled with rabbit anti-Pfs230 antisera (red). Nuclei were highlighted with Hoechst33342 nuclear stain (blue). Bar, 5 μm. Results (in B and C) are representative of three independent experiments.
(TIF)

S7 Fig Subcellular fractioning of 7-Helix-1-HA gametocytes and co-localization with the osmiophilic body protein Pfg377. (A) Gametocyte lysates of the 7-Helix-1-HA line were used to extract soluble, integral and insoluble protein fractions. The samples were subjected to WB and immunolabeled with rabbit anti-HA antibodies to detect 7-Helix-1-HA (~60 kDa), mouse anti-Falcilysin antisera (~140 kDa), rabbit anti-CCp2 antisera (~185 kDa) and mouse anti-*Pf*s16 antisera (~16 kDa). Results are representative of three independent experiments. (B) Co-localization analysis of 7-Helix-1 with the osmiophilic body protein Pfg377. Gametocytes were immunolabeled with mouse anti-7-Helix-1rp1 and anti-7-Helix-1rp2 antisera (green) and rabbit anti-Pfg377 antisera (red). Nuclei were highlighted with Hoechst33342 nuclear stain (blue). Bar, 5 μm. Results are representative of five independent experiments.
(TIF)

S8 Fig Generation of the 7-Helix-1-KO lines. (A) Schematic depicting the single-crossover homologous recombination strategy for *7-helix-1* disruption. The numbered arrows indicate positions of primers used to confirm integration of the 7-Helix-1-KO pCAM-BSD vector. (B) Confirmation of gene locus integration of the 7-Helix-1-KO pCAM-BSD vector. Diagnostic PCR was used to demonstrate vector integration in the 1D12 and 2E6 lines. Primers 1 and 4 were used to demonstrate 5‘-integration and primers 2 and 3 were used to demonstrate 3‘-integration. Primers 3 and 4 were used to detect the episomal plasmid, while primers 1 and 2 were used for amplification of the WT *7-helix-1* locus. The gDNA of a WT NF54 was used as a negative control. (C) Expression analysis of 7-Helix-1 in the 7-Helix-1-KO lines. Mature gametocytes of WT NF54 and the 7-Helix-1-KO lines 1D12 and 2E6 were immunolabeled with mouse anti-7-Helix-1rp1 antisera (green) and counterlabeled with rabbit anti-Pfs230 antisera (red). Nuclei were highlighted with Hoechst33342 nuclear stain (blue). Bar, 5 μm. (D) Confirmation of lack of 7-Helix-1 in the 7-Helix-1-KO lines. Gametocyte (GC) lysates of WT NF54 and the two 7-Helix-1-KO lines 1D12 and 2E6 were subjected to WB and immunolabeled with mouse anti-7-Helix-1rp2 antisera to detect 7-Helix-1 (~50 kDa). Equal loading was confirmed using a polyclonal mouse anti-Pf39 antiserum (~39 kDa). Results (in B-D) are representative of three independent experiments.
(TIF)

S9 Fig Sequencing of the integration locus of the 7-Helix-1-KO line 2E6. (A) Sequencing of the 5‘ integration locus. (B) Sequencing of the 3‘ integration locus. The sequences corresponding to the WT NF54 *7-helix-1* locus are indicated with green letters; sequences corresponding to the vector backbone of the pCAM-BSD vector are indicated with red letters. Primers used for the generation of the pCAM-BSD-7-Helix-1-KO construct are indicated with orange letters. Sequences are shown in 5‘-3‘-orientation.
(TIF)

S10 Fig Phenotype analysis of 7-Helix-1-KO parasites during erythrocytic replication. (A) Morphology of the 7-Helix-1-KO asexual blood stages. Giemsa smears of ring stages (R), trophozoites (TZ), immature (imSZ) and mature (mSZ) schizonts of WT NF54 and the 7-Helix-1-KO lines 2E6 and 1D12 were microscopically analyzed. Bar, 5 μm. (B) Quantification of the asexual blood stages during erythrocytic replication. The numbers of Giemsa-stained asexual blood stages as indicated above were counted at seven time points over a period of 49 h. A total number of 50 parasites per time point was counted in triplicate (mean ± SD). Results are representative for three independent experiments.
(TIF)

S11 Fig Phenotype analysis of 7-Helix-1-KO parasites during gametocyte development. (A) Morphology of 7-Helix-1-KO gametocytes. Giemsa smears of gametocytes of stages II to V (GCII—GCV) of WT NF54 and the 7-Helix-1-KO lines 2E6 and 1D12 were microscopically analyzed. Bar, 5 μm. (B) Quantification of the gametocyte stages during maturation. The numbers of Giemsa-stained gametocytes as indicated above were counted at six time points over a period of 15 d. A total number of 50 parasites per time point was counted in triplicate (mean ± SD). Results are representative for three independent experiments.
(TIF)

S12 Fig Generation of the 7-Helix-1-KO(+) complementation line. (A) Verification of the 7-Helix-1-KO(+) complementation line at the DNA level. Diagnostic PCR was employed, using DNA isolated from the 7-Helix-1-KO(+) line and from WT NF54 as templates. Amplification of a 189 bp sequence of *pfama1* was used as a gDNA control. Expected size of the pARL2-FNPA5‘-7-Helix-1-GFP episome amplification product, 831 bp. (B) Verification of the 7-Helix-1-KO(+) complementation line at the protein level. Gametocyte (GC) lysates of WT NF54, the 7-Helix-1-KO clone 1D12 and the complementation line (7-Helix-1-KO(+)) were subjected to WB and immunolabeled with mouse anti-7-Helix-1rp2 antisera to detect 7-Helix-1 (~50 kDa; asterisk). The presence of 7-Helix-1-GFP (~70 kDa; arrow) was further confirmed by immunoblotting with rabbit anti-GFP antibody. Equal loading was confirmed using a polyclonal mouse anti-Pf39 antiserum (~39 kDa). (C,D) Expression analysis of 7-Helix-1-GFP in gametocytes. Gametocytes of WT NF54 and the 7-Helix-1-KO(+) line were immunolabeled with mouse anti-7-Helix-1rp1 (C) and anti-7-Helix-1rp2 antisera (D) (green) and counterlabeled with rabbit anti-Pfs230 antisera (red). Nuclei were highlighted with Hoechst33342 nuclear stain (blue). Bar, 5 μm. Results are representative of three independent experiments.
(TIF)

S13 Fig Analysis of transcript level changes for selected genes in 7-Helix-1-KO gametocytes. (A) Transcript level analysis of gametocytes at 30’ p.a. for five genes with up-regulated transcript levels and two genes with down-regulated transcript levels as identified by microarray. (B) Transcript level analysis of *sr1*, *sr10*, *sr12* and *sr25* in mature gametocytes (black bars) and gametocytes at 30’ p.a. (grey bars). Transcript levels were calculated for WT and 7-Helix-1-KO line 2E6 gametocytes by the 2^-ΔCt^ method; the Ct was normalized with the Ct of the gene encoding seryl tRNA-ligase (PF3D7_0717700) as reference and fold changes between 7-Helix-1-KO and WT were calculated. Measurements were performed in triplicate (mean ± SD). Results are representative of three independent experiments.
(TIF)

S14 Fig Co-localization analysis of 7-Helix-1 with SGs. Mature WF NF54 gametocytes (A, D), 7-Helix-1-HA gametocytes (B, E) or RNF1-HA gametocytes (C) were subjected to mRNA-FISH-IFA. (A) Negative control lacking the poly-dT-oligonucleotides; 7-Helix-1 was labelled with anti-7-Helix-1rp2 (green). (B-E) Labeling of mRNA with a biotinylated oligo-dT25 probe (red); counterlabeling was performed using rabbit anti-HA antibodies (B, C) or mouse anti-Pfs230 antisera (D, E) (green). Nuclei (in A-E) were highlighted by Hoechst33342 nuclear stain (blue). DIC, differential interference contrast. Bar, 5 μm. Results are representative of three independent experiments.
(TIF)

S15 Fig Expression analysis of Puf2 in WT and 7-Helix-1-KO gametocytes. (A) Schematic depicting the domain structure of Puf2. Pumilio-family RNA binding repeats are highlighted with orange boxes. The black bar underneath the structure denotes the region of the recombinant protein. AA, amino acids. (B-D) Immunolocalization of Puf2 in gametocytes. Schizonts (SZ) and gametocytes (GC) of WT NF54 (B, C) and 7-Helix-1-KO 2E6 gametocytes (D) were immunolabeled with mouse anti-Puf2 antisera (green) and counterlabeled with rabbit antisera directed against MSP1, Pfs230 or Pfs25 (red). Nuclei (in B-D) were highlighted by Hoechst33342 nuclear stain (blue,). Bar, 5 μm. Results (in B-D) are representative of three independent experiments.
(TIF)

S16 Fig Purity controls. (A) RT-PCR control for possible contamination with gDNA. The cDNA templates to be used for diagnostic RT-PCR of rings (R), trophozoites (TZ), schizonts (SZ), immature (GCII-IV), mature (GCV) and activated (aGC) gametocytes were prepared without adding reverse transcriptase. Diagnostic PCR was performed with *pfaldolase*-specific primers. Expected size of PCR product, 378 bp. (B) RT-PCR to control purity of cDNA isolated from WT NF54 and 7-Helix-1-KO 2E6 gametocytes at 30’ p.a. used for real time RT-PCR. Transcript analyses of *pfama1* (189 bp; specific for asexual stages) and *pfccp2* (198 bp; specific for gametocyte stages) were used to demonstrate purity of the gametocyte samples. Transcript analysis of *pfaldolase* (378 bp) was used for loading control. To exclude contamination of the samples with genomic DNA, cDNA samples were prepared lacking reverse transcriptase (-RT) and PCR was performed using *pfaldolase*-specific primers. Expected size of PCR product, 378 bp. (C) Control of purity of gametocyte samples used for microarray analyses. Purity of Percoll-purified mature gametocytes of WT NF54 and the 7-Helix-1-KO 2E6 line was determined by Giemsa-stained smears.
(TIF)

S1 Table Mean no. of salivary gland sporozoites following SMFAs. (DOCX)

S2 Table List of primers used in this study. (DOCX)

S1 File List of genes with deregulated transcripts in 7-Helix-1-KO gametocytes at 30 min p.a. Genes as identified by microarray analysis, raw data.
(XLSX)

S2 File List of genes with deregulated transcripts in 7-Helix-1-KO gametocytes at 30 min p.a. Genes as identified by microarray analysis, fold-changes, grouped by function.
(XLSX)

S3 File GO enrichment analysis of deregulated transcripts in 7-Helix-1-KO gametocytes at 30 min p.a. (XLSX)
