# Supplementary figures and images for: A seven-helix protein constitutes stress granules crucial for regulating translation during human-to-mosquito transmission of Plasmodium falciparum
Source: PLoS Pathog. 2018 Aug 22;14(8):e1007249. doi: 10.1371/journal.ppat.1007249 (PMC6122839; doi:10.1371/journal.ppat.1007249)

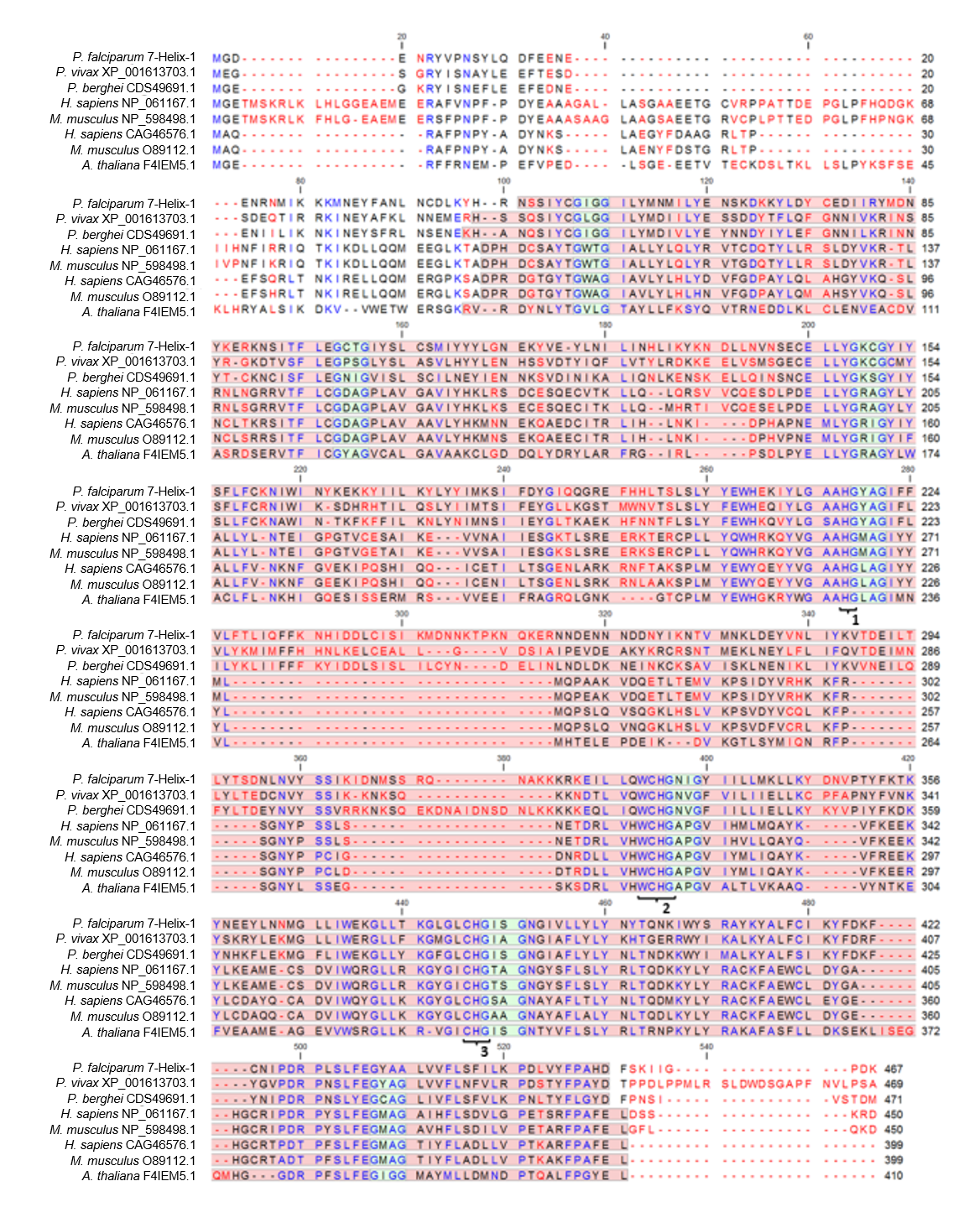

Supplement: S1 Fig — Amino acid sequence alignment of 7-Helix-1 and related proteins from other organisms generated with CLC MainWorkbench 7.5. The degree of conservation of the single amino acids is shown in color codes (0–30%, red; 30–60%, black; 60–100%, blue). The conserved LanCL domain was identified with the Simple Modular Architecture Research Tool-SMART and is highlighted in red. The seven conserved GXXG motifs are highlighted in green. The highly conserved motifs HG in repeat 4 (1), WCXG in repeat 5 (2) and CHG in repeat 6 (3) are labelled with brackets. (TIF) [file ppat.1007249.s001.tif]

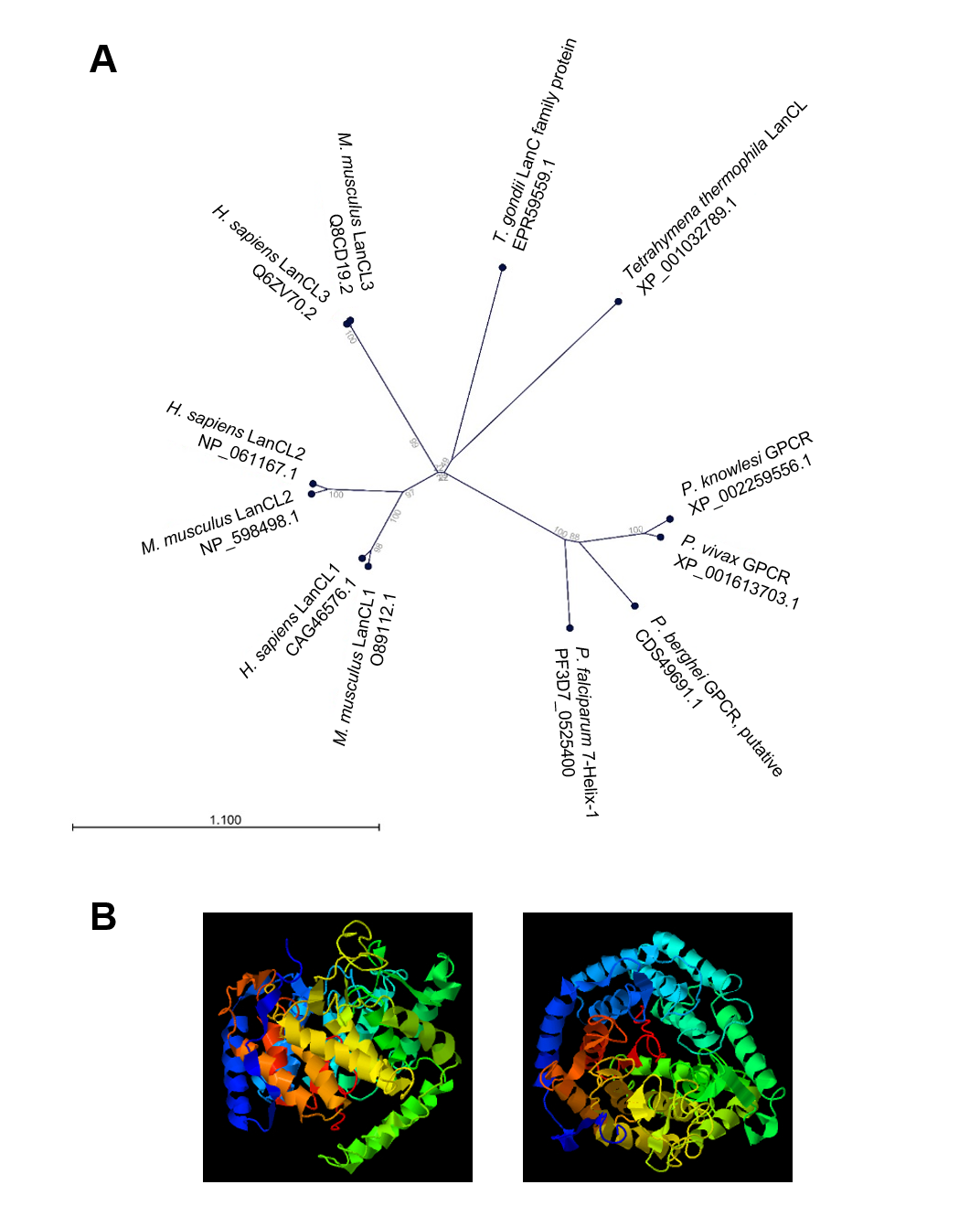

Supplement: S2 Fig — (A) Phylogenetic tree of 7-Helix-1. Phylogenetic analysis was performed using CLC MainWorkbench 7.5. The values on the branching points of the tree are corresponding to the relative consensus during 1000 bootstraps. (B) Modelling of the 3D structure of 7-Helix-1. The 3D structure was generated based on the amino acid sequence and the crystal structure of hLanCL1 using the I-TASSER server. The sequence is shown from the N-terminus (blue) to the C-terminus (red). Left panel, side view; right panel, top view. (TIF) [file ppat.1007249.s002.tif]

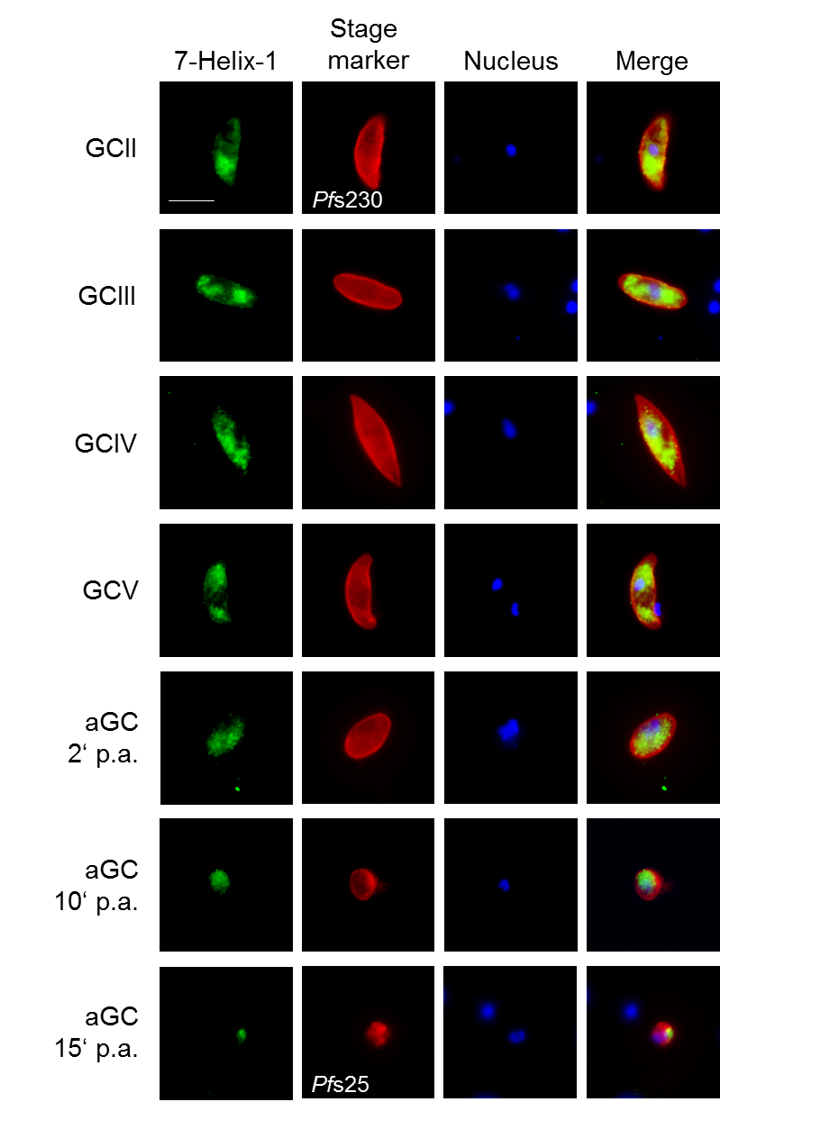

Supplement: S3 Fig — Gametocytes (GC stage II–V) and activated gametocytes (aGC; at 2, 10 and 15 min p.a.) of WT NF54 were immunolabeled with mouse anti- 7-Helix-1rp2 antisera (green) and counterlabeled with rabbit antibodies directed against Pfs230 and Pfs25 (red). Nuclei were highlighted with Hoechst33342 nuclear stain (blue). Bar, 5 μm. Results are representative of five independent experiments. (TIF) [file ppat.1007249.s003.tif]

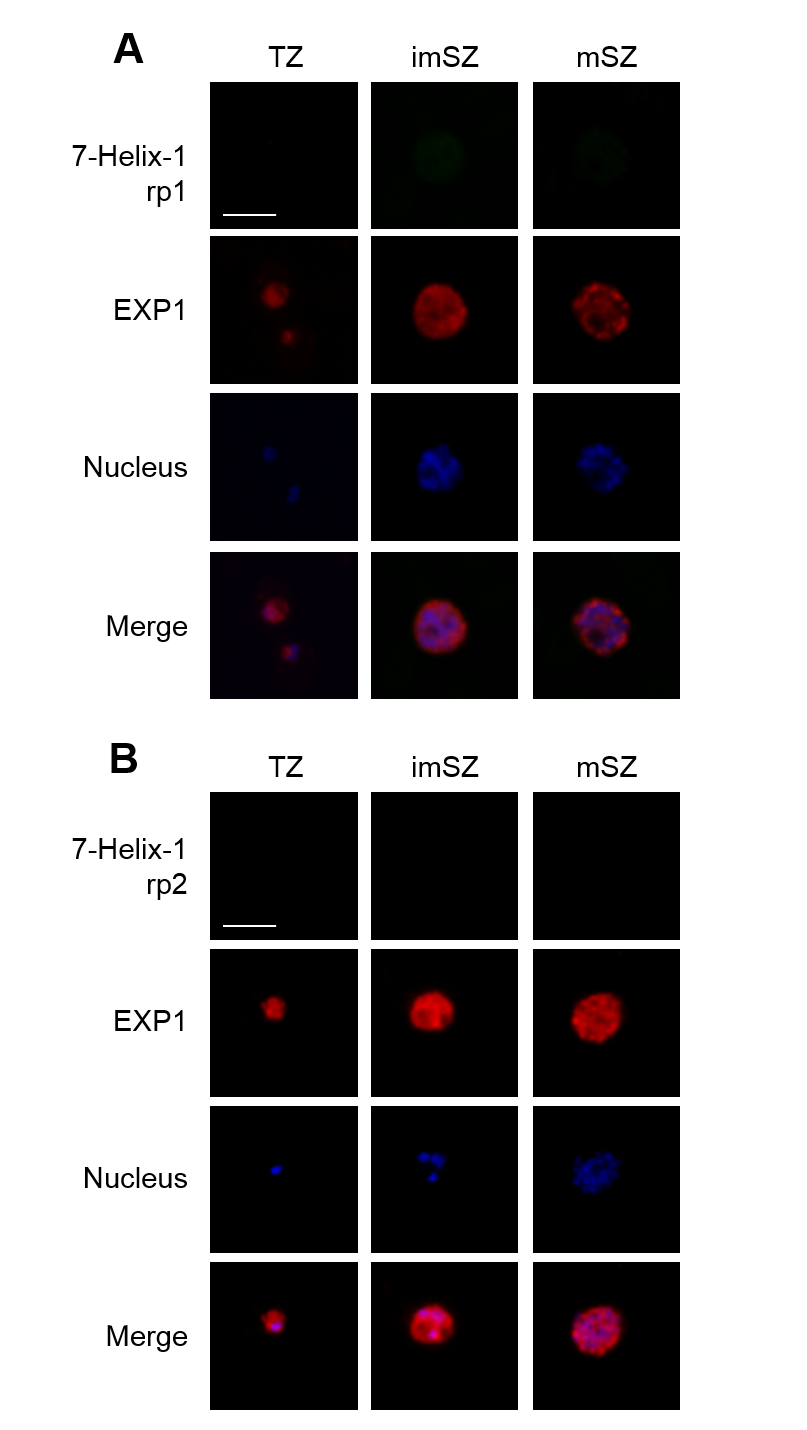

Supplement: S4 Fig — Trophozoites (TZ), immature (imSZ) and mature (mSZ) schizonts of WT NF54 were immunolabeled with mouse anti-7-Helix-1rp1 (A) or anti-7-Helix-1rp2 (B) antisera (green) and counterlabeled with rabbit anti-EXP1 antibody (red). Nuclei (in A and B) were highlighted with Hoechst33342 nuclear stain (blue). Bar, 5 μm. Results are representative of five independent experiments. (TIF) [file ppat.1007249.s004.tif]

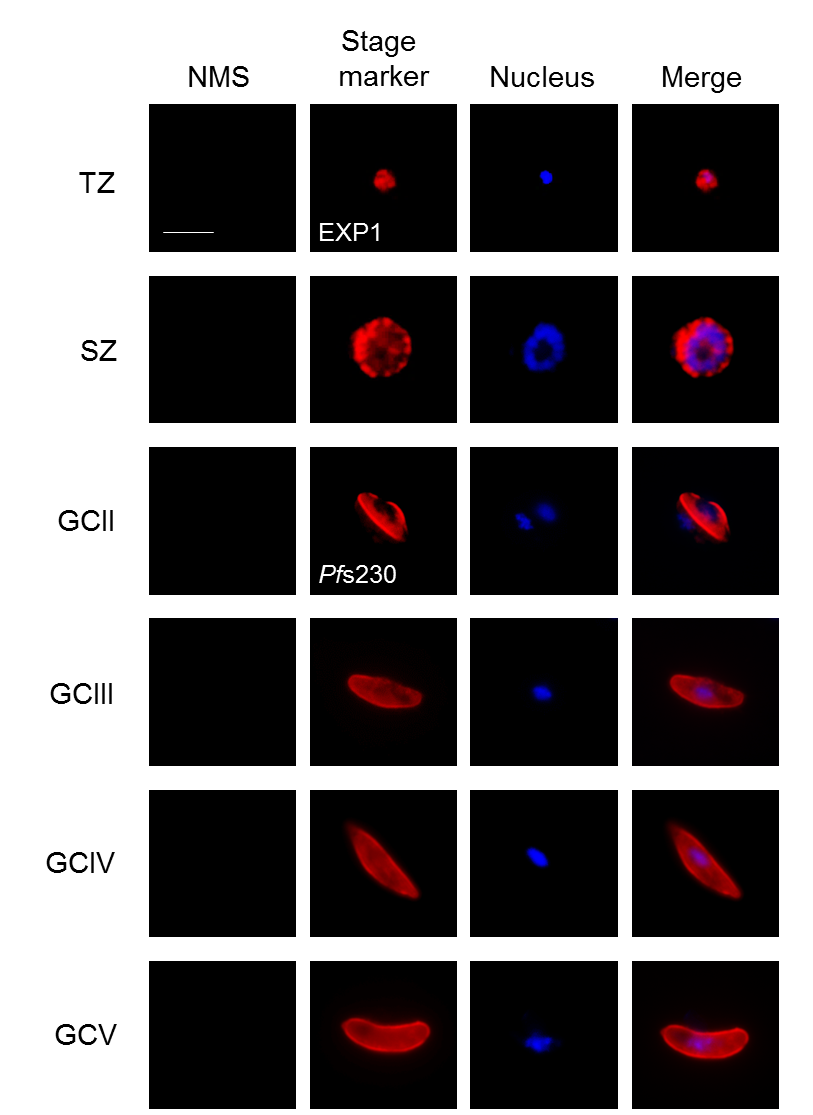

Supplement: S5 Fig — Trophozoites (TZ), schizonts (SZ) and gametocytes of stages II-V (GCII—GCV) of WT NF54 were incubated with NMS (green) and counterlabeled with rabbit antibodies directed against-EXP1 and Pfs230 as indicated (red). Nuclei were highlighted with Hoechst33342 nuclear stain (blue). Bar, 5 μm. Results are representative of five independent experiments. (TIF) [file ppat.1007249.s005.tif]

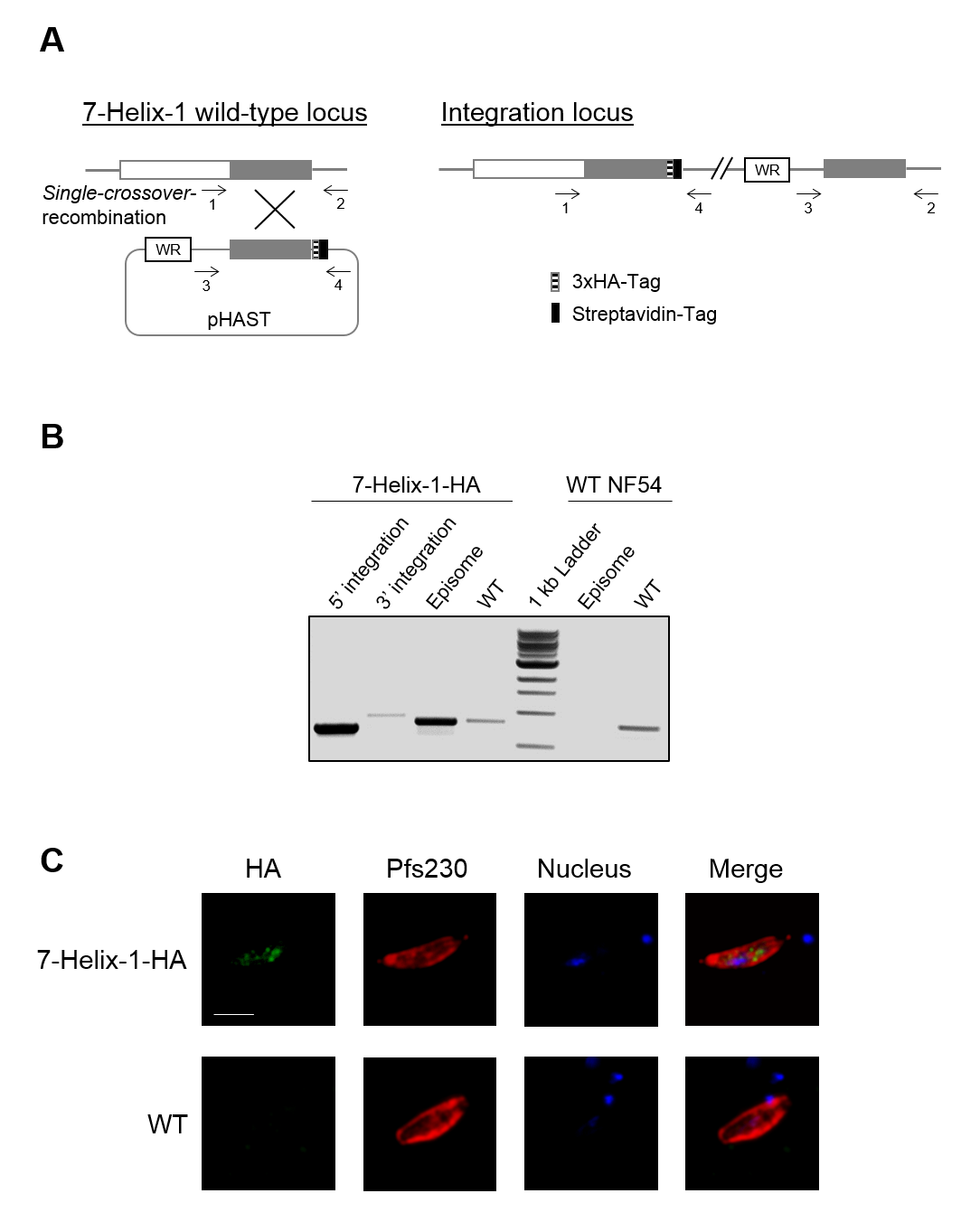

Supplement: S6 Fig — (A) Schematic depicting the single-crossover homologous recombination strategy for 7-helix-1 fusion to the 3xHA-Streptavidin-tag sequence. The numbered arrows indicate positions of primers used to confirm integration of the 7-Helix-1-HA-pHAST vector. (B) Confirmation of gene locus integration of the 7-Helix-1-HA-pHAST vector. Diagnostic PCR was used to demonstrate vector integration. Primers 1 and 4 were used to demonstrate 5‘-integration and primers 2 and 3 were used to demonstrate 3‘-integration. Primers 3 and 4 were used to detect the episomal plasmid, while primers 1 and 2 were used for amplification of the WT 7-helix-1 locus. The gDNA of a WT NF54 was used as a negative control. (C) Localization of 7-Helix-1 in the 7-Helix-1-HA line. Mature gametocytes of WT NF54 and the 7-Helix-1-HA line were immunolabeled with rabbit anti-HA antibodies (green) and counterlabeled with rabbit anti-Pfs230 antisera (red). Nuclei were highlighted with Hoechst33342 nuclear stain (blue). Bar, 5 μm. Results (in B and C) are representative of three independent experiments. (TIF) [file ppat.1007249.s006.tif]

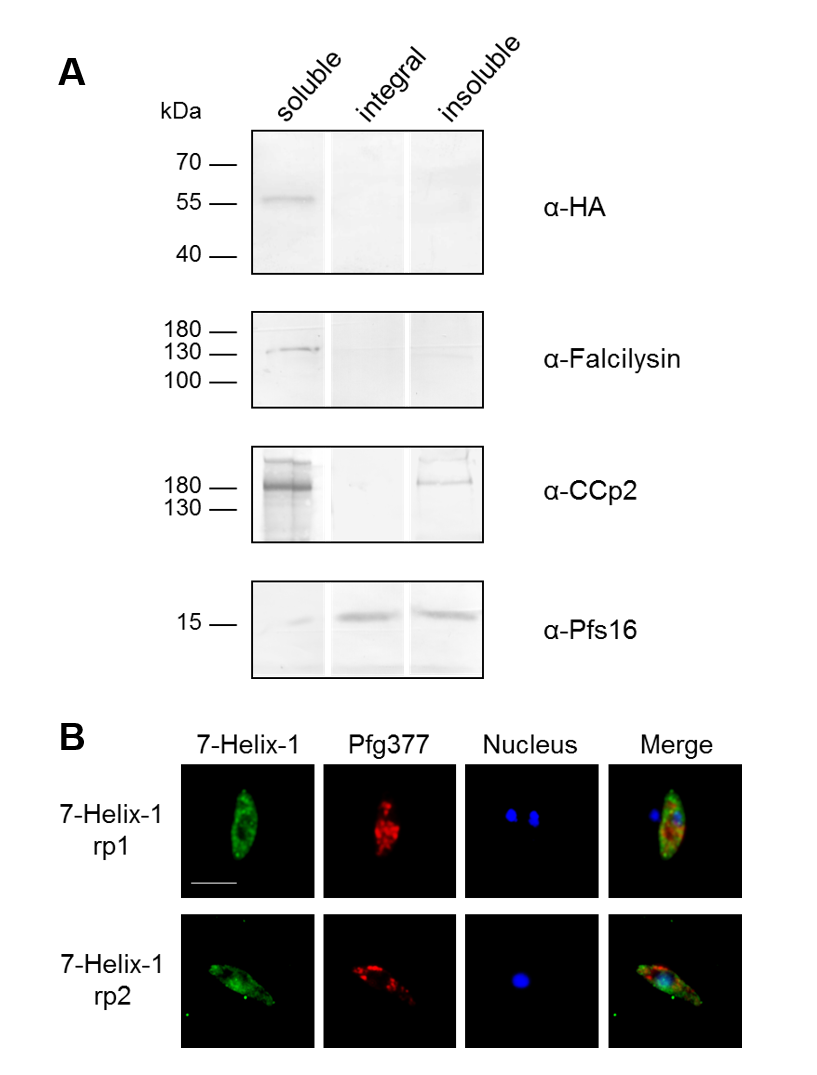

Supplement: S7 Fig — (A) Gametocyte lysates of the 7-Helix-1-HA line were used to extract soluble, integral and insoluble protein fractions. The samples were subjected to WB and immunolabeled with rabbit anti-HA antibodies to detect 7-Helix-1-HA (~60 kDa), mouse anti-Falcilysin antisera (~140 kDa), rabbit anti-CCp2 antisera (~185 kDa) and mouse anti-Pfs16 antisera (~16 kDa). Results are representative of three independent experiments. (B) Co-localization analysis of 7-Helix-1 with the osmiophilic body protein Pfg377. Gametocytes were immunolabeled with mouse anti-7-Helix-1rp1 and anti-7-Helix-1rp2 antisera (green) and rabbit anti-Pfg377 antisera (red). Nuclei were highlighted with Hoechst33342 nuclear stain (blue). Bar, 5 μm. Results are representative of five independent experiments. (TIF) [file ppat.1007249.s007.tif]

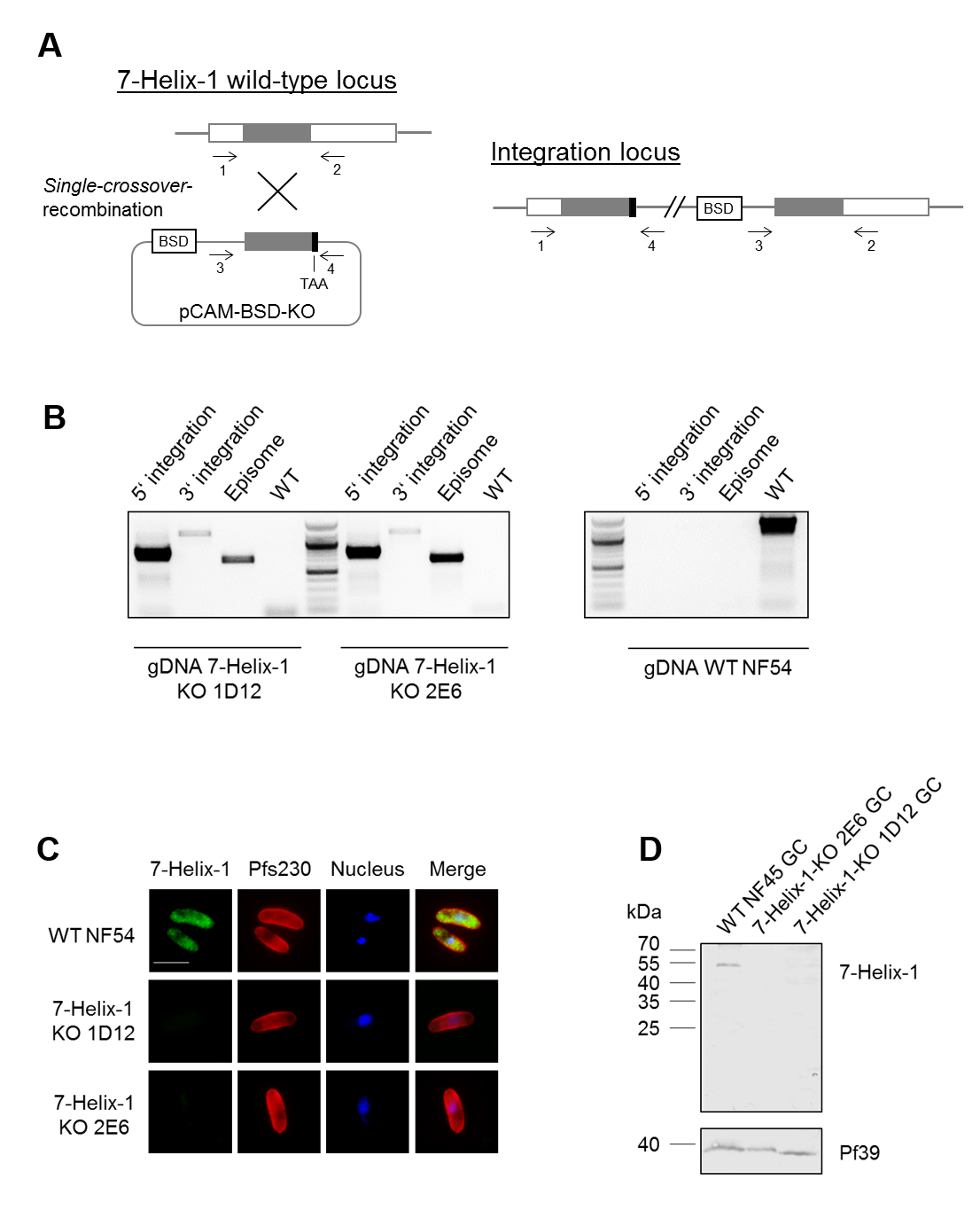

Supplement: S8 Fig — (A) Schematic depicting the single-crossover homologous recombination strategy for 7-helix-1 disruption. The numbered arrows indicate positions of primers used to confirm integration of the 7-Helix-1-KO pCAM-BSD vector. (B) Confirmation of gene locus integration of the 7-Helix-1-KO pCAM-BSD vector. Diagnostic PCR was used to demonstrate vector integration in the 1D12 and 2E6 lines. Primers 1 and 4 were used to demonstrate 5‘-integration and primers 2 and 3 were used to demonstrate 3‘-integration. Primers 3 and 4 were used to detect the episomal plasmid, while primers 1 and 2 were used for amplification of the WT 7-helix-1 locus. The gDNA of a WT NF54 was used as a negative control. (C) Expression analysis of 7-Helix-1 in the 7-Helix-1-KO lines. Mature gametocytes of WT NF54 and the 7-Helix-1-KO lines 1D12 and 2E6 were immunolabeled with mouse anti-7-Helix-1rp1 antisera (green) and counterlabeled with rabbit anti-Pfs230 antisera (red). Nuclei were highlighted with Hoechst33342 nuclear stain (blue). Bar, 5 μm. (D) Confirmation of lack of 7-Helix-1 in the 7-Helix-1-KO lines. Gametocyte (GC) lysates of WT NF54 and the two 7-Helix-1-KO lines 1D12 and 2E6 were subjected to WB and immunolabeled with mouse anti-7-Helix-1rp2 antisera to detect 7-Helix-1 (~50 kDa). Equal loading was confirmed using a polyclonal mouse anti-Pf39 antiserum (~39 kDa). Results (in B-D) are representative of three independent experiments. (TIF) [file ppat.1007249.s008.tif]

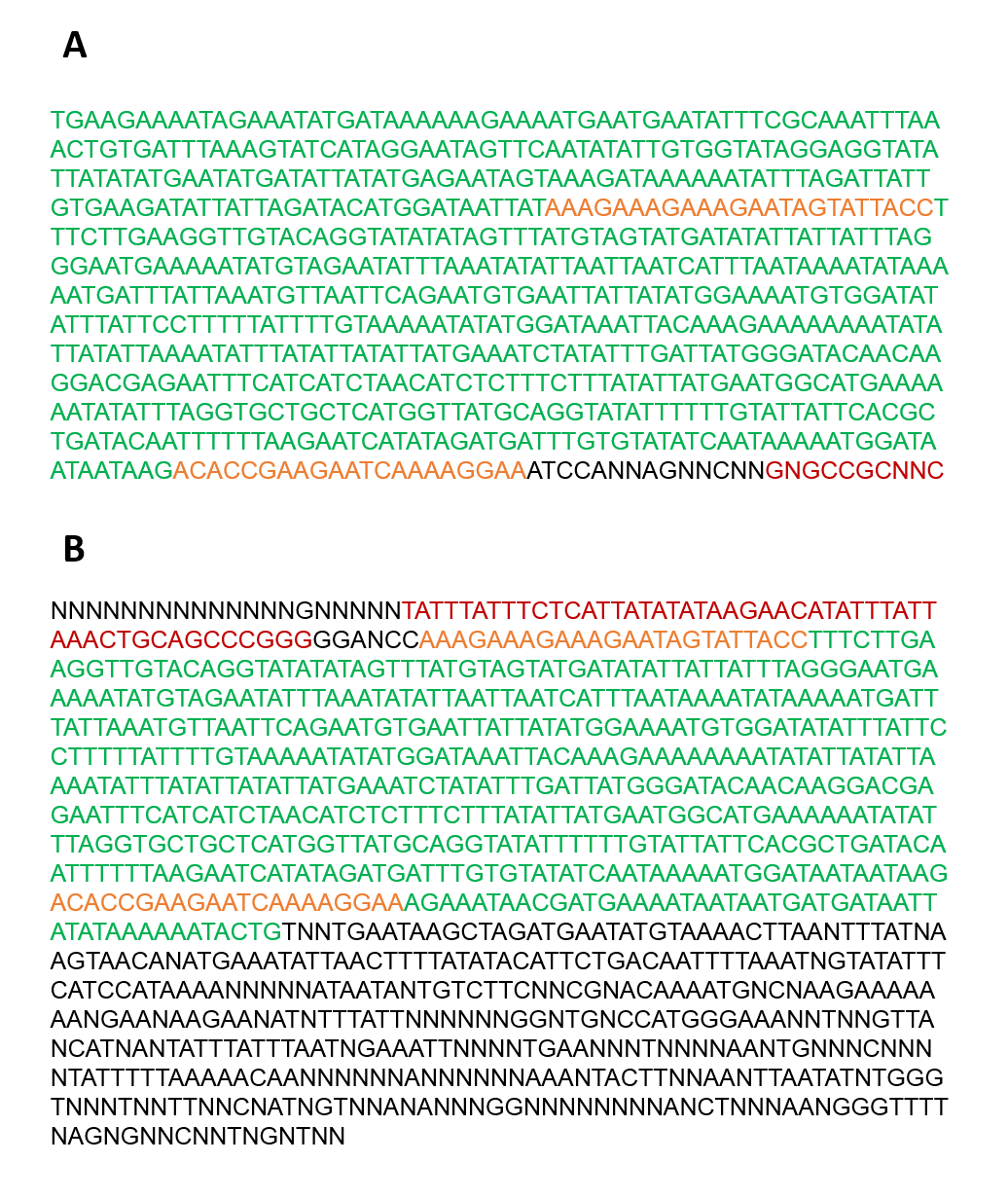

Supplement: S9 Fig — (A) Sequencing of the 5‘ integration locus. (B) Sequencing of the 3‘ integration locus. The sequences corresponding to the WT NF54 7-helix-1 locus are indicated with green letters; sequences corresponding to the vector backbone of the pCAM-BSD vector are indicated with red letters. Primers used for the generation of the pCAM-BSD-7-Helix-1-KO construct are indicated with orange letters. Sequences are shown in 5‘-3‘-orientation. (TIF) [file ppat.1007249.s009.tif]

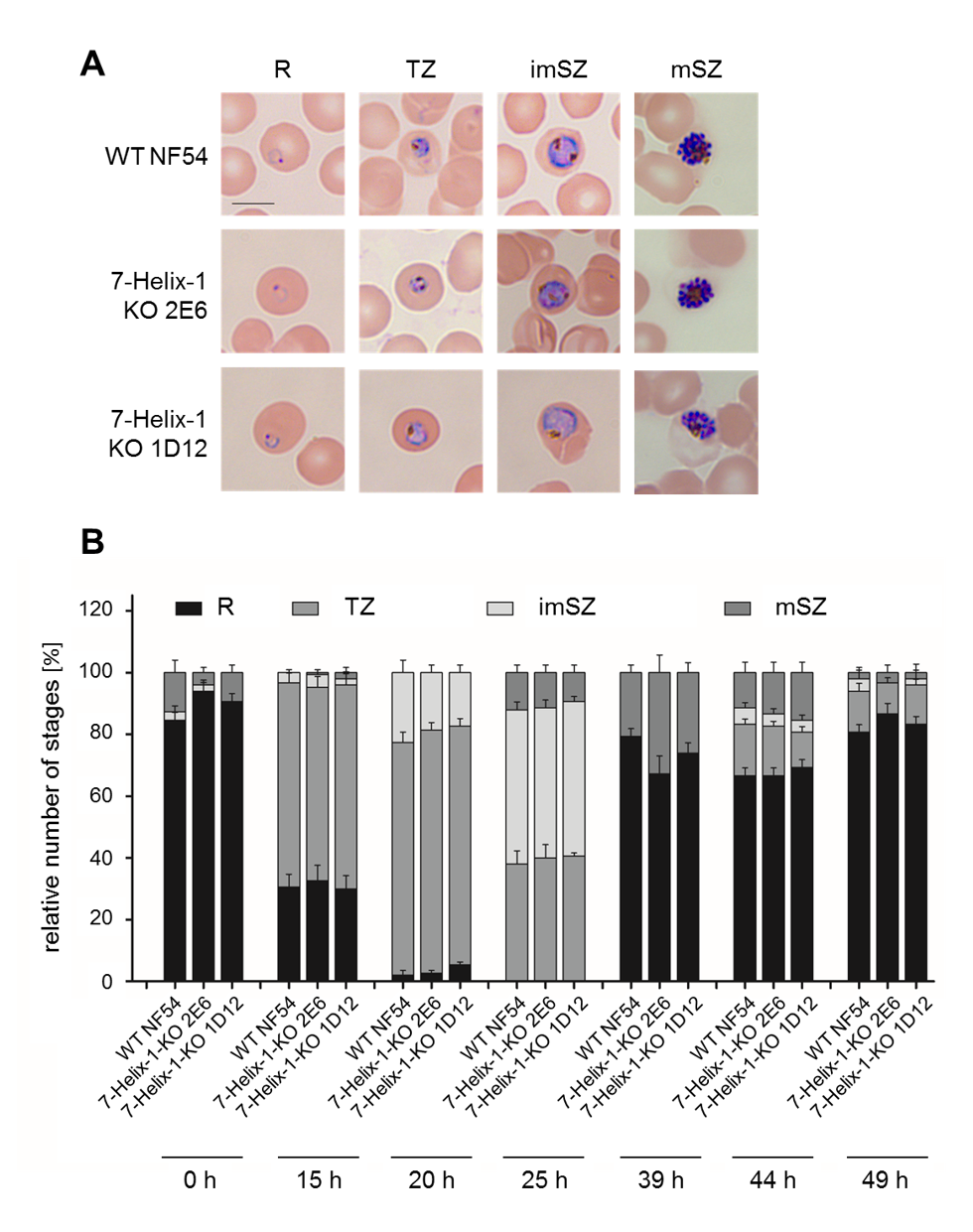

Supplement: S10 Fig — (A) Morphology of the 7-Helix-1-KO asexual blood stages. Giemsa smears of ring stages (R), trophozoites (TZ), immature (imSZ) and mature (mSZ) schizonts of WT NF54 and the 7-Helix-1-KO lines 2E6 and 1D12 were microscopically analyzed. Bar, 5 μm. (B) Quantification of the asexual blood stages during erythrocytic replication. The numbers of Giemsa-stained asexual blood stages as indicated above were counted at seven time points over a period of 49 h. A total number of 50 parasites per time point was counted in triplicate (mean ± SD). Results are representative for three independent experiments. (TIF) [file ppat.1007249.s010.tif]

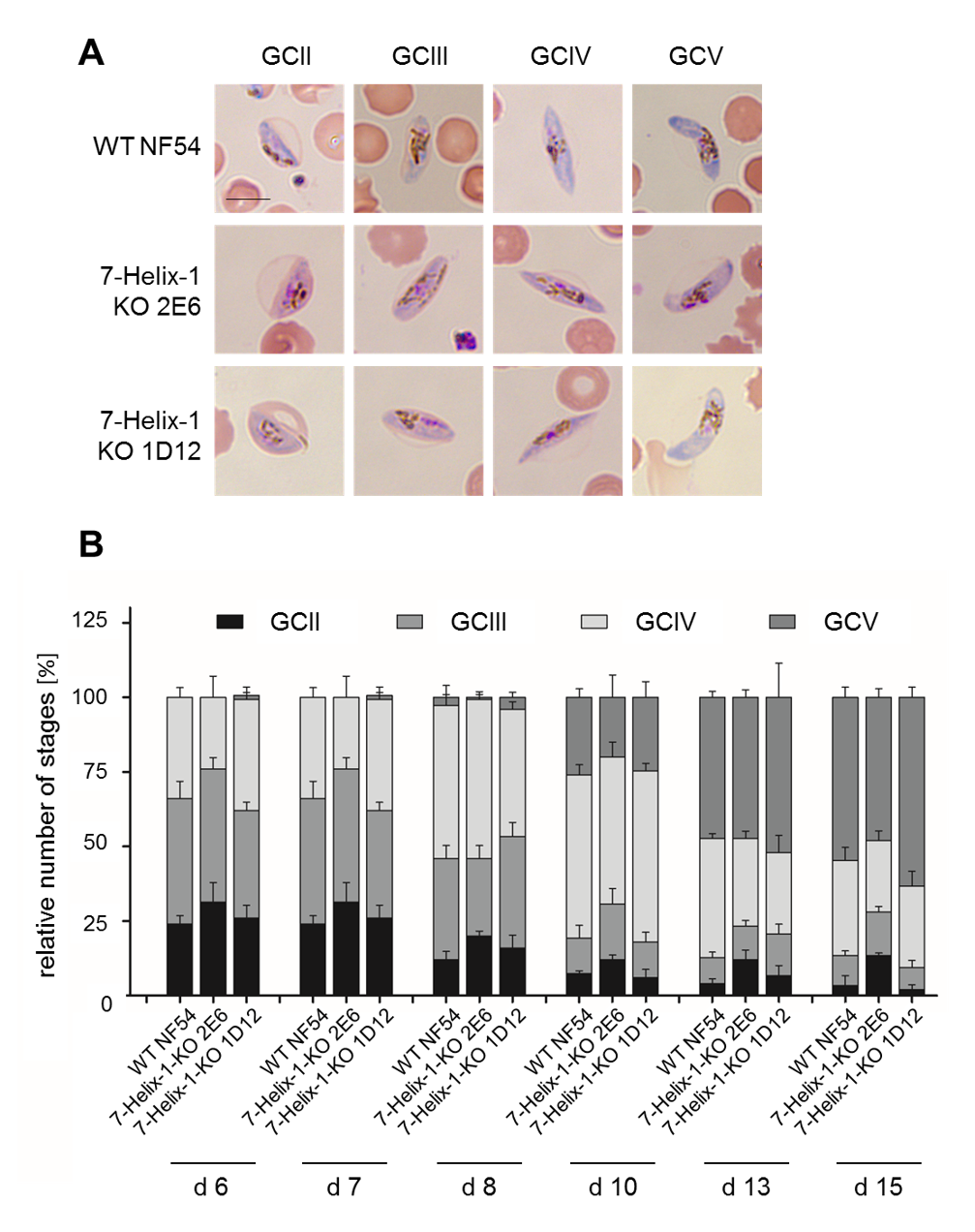

Supplement: S11 Fig — (A) Morphology of 7-Helix-1-KO gametocytes. Giemsa smears of gametocytes of stages II to V (GCII—GCV) of WT NF54 and the 7-Helix-1-KO lines 2E6 and 1D12 were microscopically analyzed. Bar, 5 μm. (B) Quantification of the gametocyte stages during maturation. The numbers of Giemsa-stained gametocytes as indicated above were counted at six time points over a period of 15 d. A total number of 50 parasites per time point was counted in triplicate (mean ± SD). Results are representative for three independent experiments. (TIF) [file ppat.1007249.s011.tif]

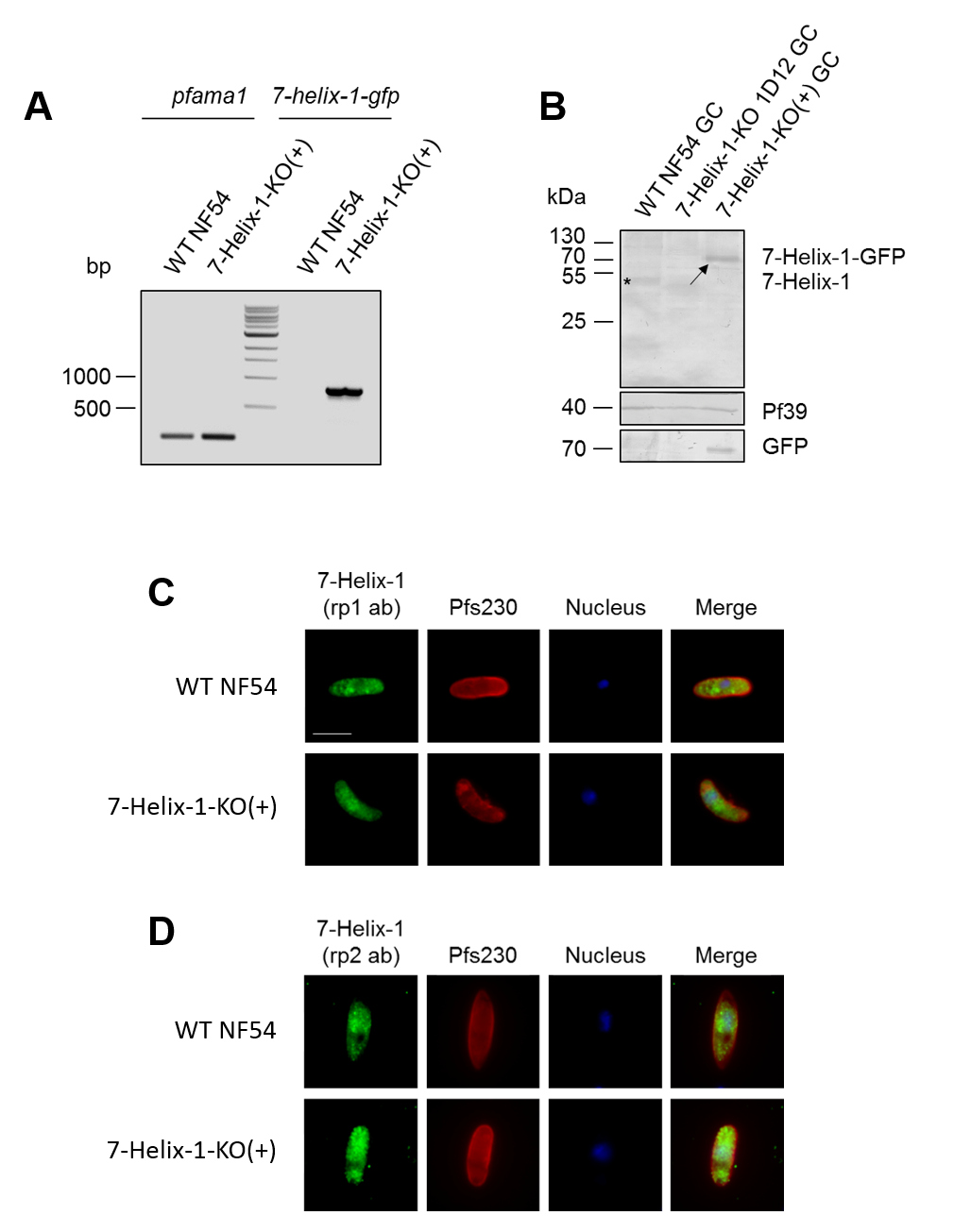

Supplement: S12 Fig — (A) Verification of the 7-Helix-1-KO(+) complementation line at the DNA level. Diagnostic PCR was employed, using DNA isolated from the 7-Helix-1-KO(+) line and from WT NF54 as templates. Amplification of a 189 bp sequence of pfama1 was used as a gDNA control. Expected size of the pARL2-FNPA5‘-7-Helix-1-GFP episome amplification product, 831 bp. (B) Verification of the 7-Helix-1-KO(+) complementation line at the protein level. Gametocyte (GC) lysates of WT NF54, the 7-Helix-1-KO clone 1D12 and the complementation line (7-Helix-1-KO(+)) were subjected to WB and immunolabeled with mouse anti-7-Helix-1rp2 antisera to detect 7-Helix-1 (~50 kDa; asterisk). The presence of 7-Helix-1-GFP (~70 kDa; arrow) was further confirmed by immunoblotting with rabbit anti-GFP antibody. Equal loading was confirmed using a polyclonal mouse anti-Pf39 antiserum (~39 kDa). (C,D) Expression analysis of 7-Helix-1-GFP in gametocytes. Gametocytes of WT NF54 and the 7-Helix-1-KO(+) line were immunolabeled with mouse anti-7-Helix-1rp1 (C) and anti-7-Helix-1rp2 antisera (D) (green) and counterlabeled with rabbit anti-Pfs230 antisera (red). Nuclei were highlighted with Hoechst33342 nuclear stain (blue). Bar, 5 μm. Results are representative of three independent experiments. (TIF) [file ppat.1007249.s012.tif]

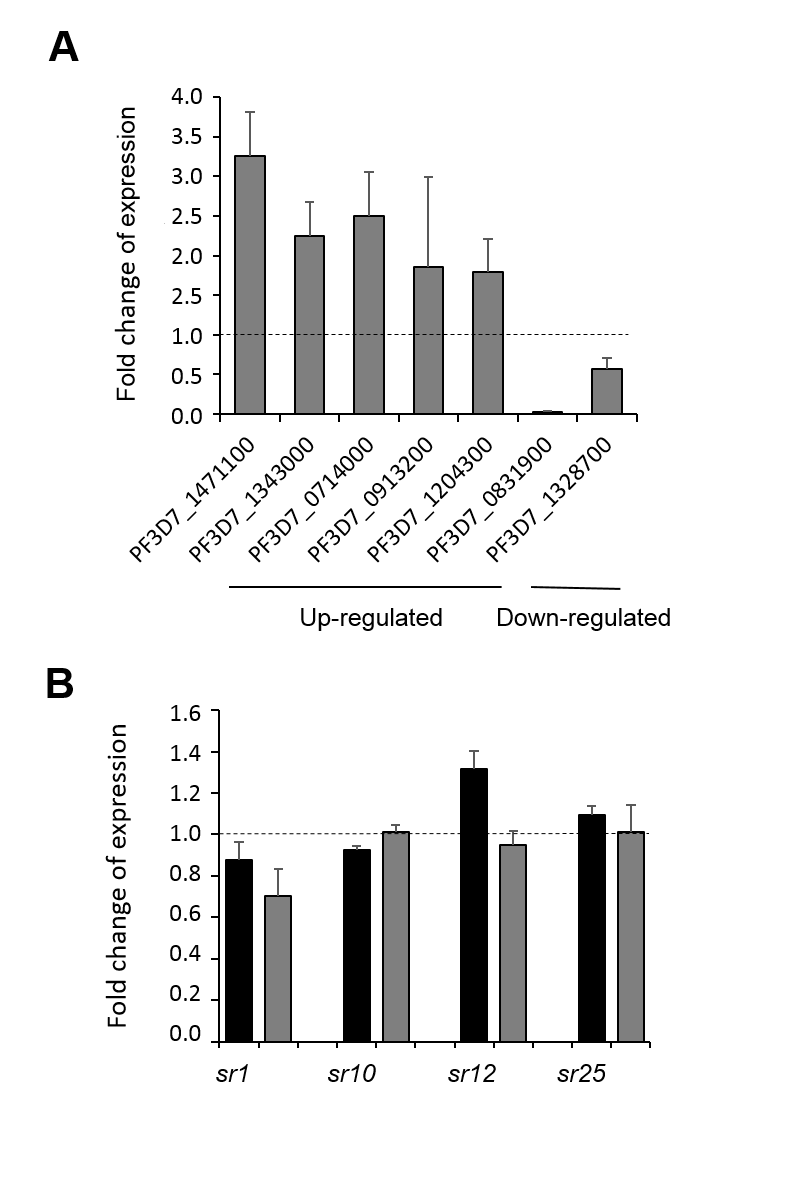

Supplement: S13 Fig — (A) Transcript level analysis of gametocytes at 30’ p.a. for five genes with up-regulated transcript levels and two genes with down-regulated transcript levels as identified by microarray. (B) Transcript level analysis of sr1, sr10, sr12 and sr25 in mature gametocytes (black bars) and gametocytes at 30’ p.a. (grey bars). Transcript levels were calculated for WT and 7-Helix-1-KO line 2E6 gametocytes by the 2-ΔCt method; the Ct was normalized with the Ct of the gene encoding seryl tRNA-ligase (PF3D7_0717700) as reference and fold changes between 7-Helix-1-KO and WT were calculated. Measurements were performed in triplicate (mean ± SD). Results are representative of three independent experiments. (TIF) [file ppat.1007249.s013.tif]

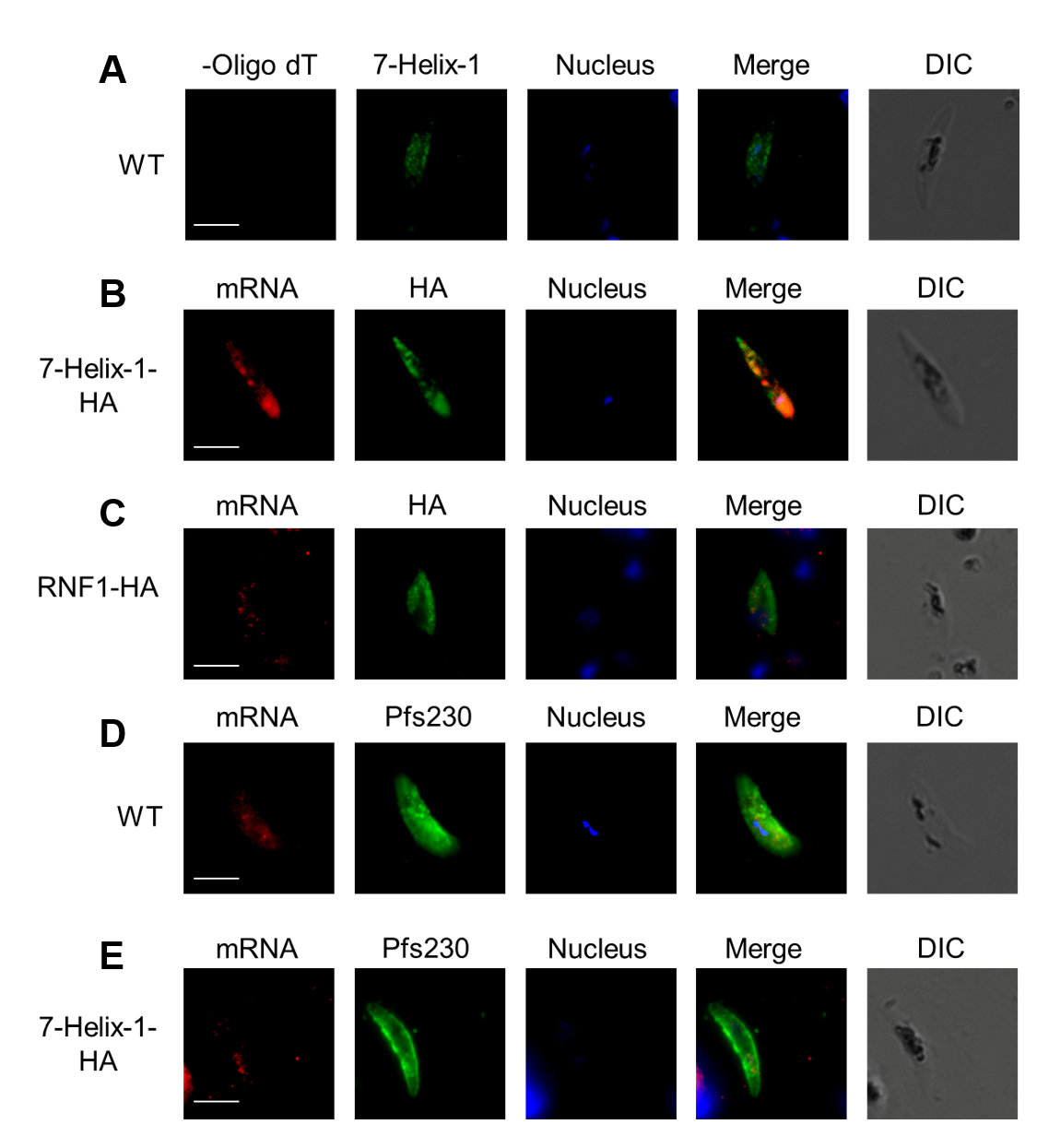

Supplement: S14 Fig — Mature WF NF54 gametocytes (A, D), 7-Helix-1-HA gametocytes (B, E) or RNF1-HA gametocytes (C) were subjected to mRNA-FISH-IFA. (A) Negative control lacking the poly-dT-oligonucleotides; 7-Helix-1 was labelled with anti-7-Helix-1rp2 (green). (B-E) Labeling of mRNA with a biotinylated oligo-dT25 probe (red); counterlabeling was performed using rabbit anti-HA antibodies (B, C) or mouse anti-Pfs230 antisera (D, E) (green). Nuclei (in A-E) were highlighted by Hoechst33342 nuclear stain (blue). DIC, differential interference contrast. Bar, 5 μm. Results are representative of three independent experiments. (TIF) [file ppat.1007249.s014.tif]

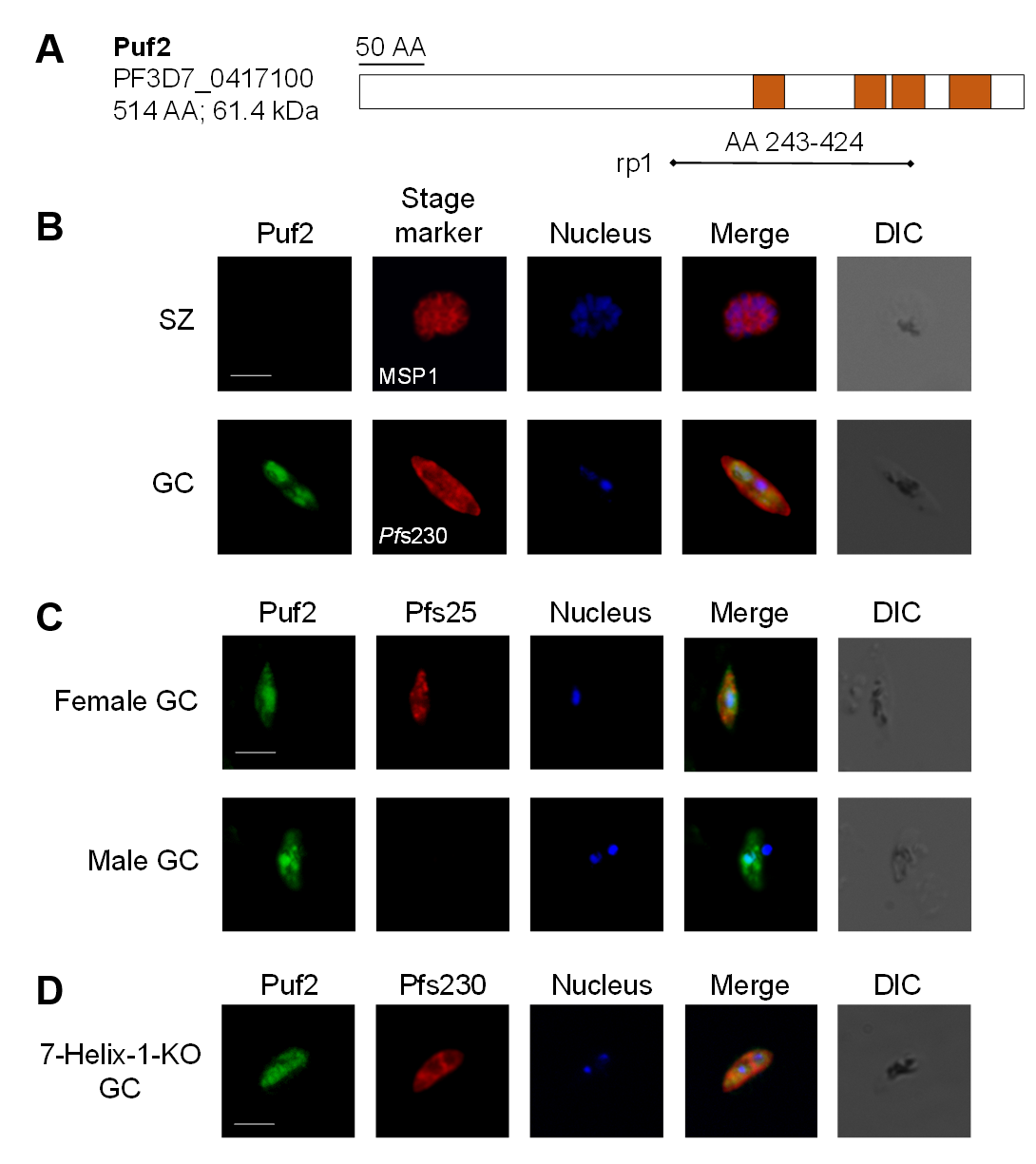

Supplement: S15 Fig — (A) Schematic depicting the domain structure of Puf2. Pumilio-family RNA binding repeats are highlighted with orange boxes. The black bar underneath the structure denotes the region of the recombinant protein. AA, amino acids. (B-D) Immunolocalization of Puf2 in gametocytes. Schizonts (SZ) and gametocytes (GC) of WT NF54 (B, C) and 7-Helix-1-KO 2E6 gametocytes (D) were immunolabeled with mouse anti-Puf2 antisera (green) and counterlabeled with rabbit antisera directed against MSP1, Pfs230 or Pfs25 (red). Nuclei (in B-D) were highlighted by Hoechst33342 nuclear stain (blue,). Bar, 5 μm. Results (in B-D) are representative of three independent experiments. (TIF) [file ppat.1007249.s015.tif]

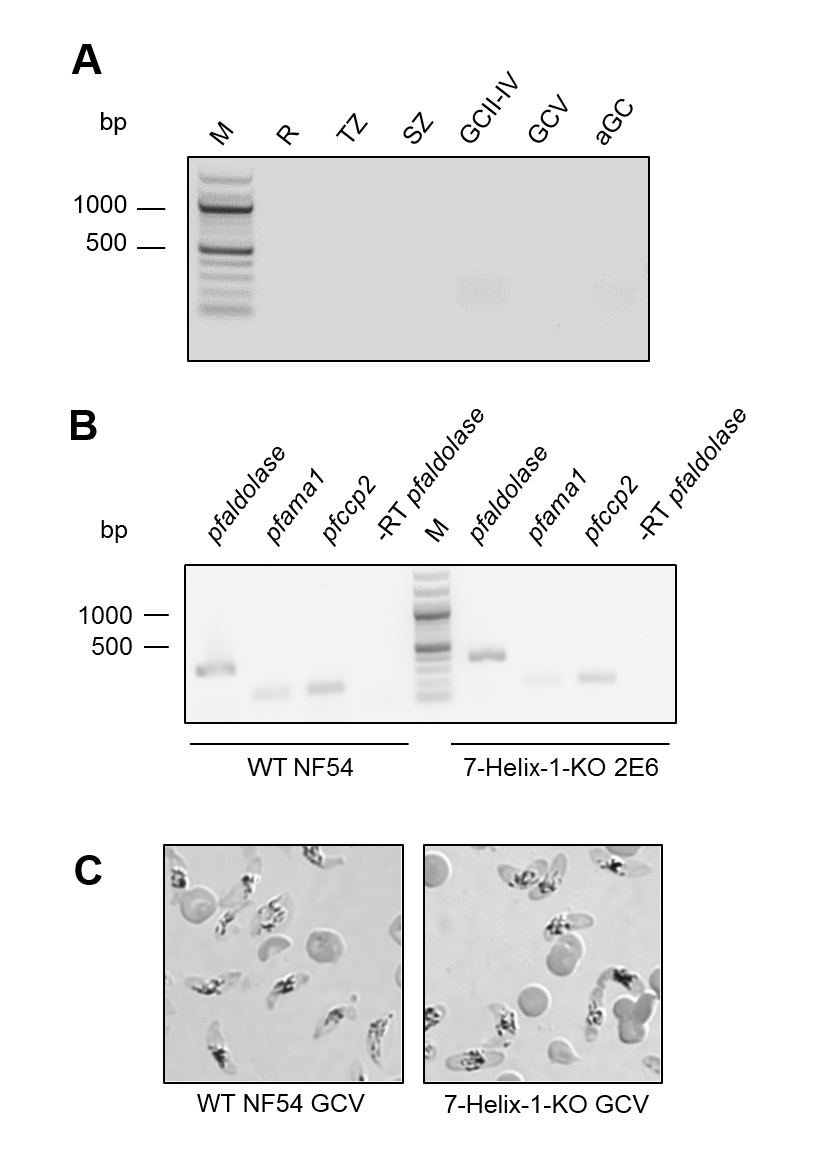

Supplement: S16 Fig — (A) RT-PCR control for possible contamination with gDNA. The cDNA templates to be used for diagnostic RT-PCR of rings (R), trophozoites (TZ), schizonts (SZ), immature (GCII-IV), mature (GCV) and activated (aGC) gametocytes were prepared without adding reverse transcriptase. Diagnostic PCR was performed with pfaldolase-specific primers. Expected size of PCR product, 378 bp. (B) RT-PCR to control purity of cDNA isolated from WT NF54 and 7-Helix-1-KO 2E6 gametocytes at 30’ p.a. used for real time RT-PCR. Transcript analyses of pfama1 (189 bp; specific for asexual stages) and pfccp2 (198 bp; specific for gametocyte stages) were used to demonstrate purity of the gametocyte samples. Transcript analysis of pfaldolase (378 bp) was used for loading control. To exclude contamination of the samples with genomic DNA, cDNA samples were prepared lacking reverse transcriptase (-RT) and PCR was performed using pfaldolase-specific primers. Expected size of PCR product, 378 bp. (C) Control of purity of gametocyte samples used for microarray analyses. Purity of Percoll-purified mature gametocytes of WT NF54 and the 7-Helix-1-KO 2E6 line was determined by Giemsa-stained smears. (TIF) [file ppat.1007249.s016.tif]
