# Supplementary material for: A seven-helix protein constitutes stress granules crucial for regulating translation during human-to-mosquito transmission of Plasmodium falciparum
Source: PLoS Pathog. 2018 Aug 22;14(8):e1007249. doi: 10.1371/journal.ppat.1007249 (PMC6122839; doi:10.1371/journal.ppat.1007249)
Supplement: S1 Table — (DOCX) [file ppat.1007249.s017.docx]

S1 Table. Mean no. of salivary gland sporozoites following SMFAs.

|  | **WT NF54** | **7-Helix-1-KO 2E6** |
| --- | --- | --- |
| SMFA 1 | 112,000 | 9,000 |
| SMFA 2 | 20,000 | 1,800 |
