# Supplementary material for: A seven-helix protein constitutes stress granules crucial for regulating translation during human-to-mosquito transmission of Plasmodium falciparum
Source: PLoS Pathog. 2018 Aug 22;14(8):e1007249. doi: 10.1371/journal.ppat.1007249 (PMC6122839; doi:10.1371/journal.ppat.1007249)
Supplement: S2 Table — (DOCX) [file ppat.1007249.s018.docx]

S2 Table. List of primers used in this study.

| PlasmoDB gene ID | Name of gene/  cloning product | Sense primer (5‘-3‘) | Anti-sense primer (5‘-3‘) |
| --- | --- | --- | --- |
| Primers used for the generation of recombinant proteins | | | |
| PF3D7_0525400 | 7-Helix-1RP1 | agaggatccAAGAATCATATAGATGATTTGTGTA | agactgcagTTTCTTGGCATTTTGTCG |
| PF3D7_0525400 | 7-Helix-1RP2 | agactggatccATGGGTGATGAAAATAGATATG | agactctcgagTTATTTGTCTGGGCCTAT |
| PF3D7_0417100 | Puf2RP1 | aaaaaaaagaaggatttcTTATGGTAGTTATGTAGCGCAAAGT | aaaaaaaactgcagGCAAGCATATTTGTGGGATGAGAG |
| PY17X_1304900 | CITH | ggccatgggcATGTCTTCTGTATCAACATTACCATATATAGGAAGTAAAATTTC | ggcctcgagCGTTGATGGCGCAATATTCATTGATACTATTGCTGG |
| Primers used for semi-quantitative RT-PCR | | | |
| PF3D7_0525400 | *7-helix-1* | GTGCTGCTCATGGTTATG | TCCATTTCCTGATATACC |
| PF3D7_1133400 | *ama1* | GGATTATGGGTCGATGGA | GATCATACTAGCGTTCTT |
| PF3D7_1455800 | *ccp2* | TCGGATGGAGAATCCGTT | GTATCCCATGTCTTGTGA |
| PF3D7_1444800 | *aldolase* | TAGATGGATTAGCAGAAAGATGC | AGAAACCAACATCTTGAGTAGTGG |
| PF3D7_1031000 | *pfs25* | AATGCGAAAGTTACCGTGGA | TCCATCAACAGCTTTACAGGT |
| PF3D7_1030900 | *pfs28* | AAGGCTCGGGTTACTGAAAA | CCTCCTCCGTTAGATGAAGGA |
| Primers used for the generation of mutant parasite lines | | | |
| PF3D7_0525400 | 7-Helix-1-KO- construct | atggatccAAAGAAAGAAAGAATAGTATTACC | tagcggccgcttaTTCCTTTTGATTCTTCGGTGT |
| PF3D7_1451600 | *fnpa* 5’-promotor region | tagcggccgcCCATACACCAACCAACCTTATTTG | tactcgagAATATGATTTATACTAAGGATTGGTATAAAG |
| PF3D7_0525400 | 7-Helix-1-KO(+)- construct | agactctcgagATGGGTGATGAAAATAGATATG | agactcctaggTTTGTCTGGGCCTATAATTTTTG |
| PF3D7_0525400 | 7-Helix-1-HA construct | agactccgcggAATACTGTTATGAATAAGCTAGATG | agactctcgagTTTGTCTGGGCCTATAAT |
| Primers used for diagnostic PCR to confirm vector integration / plasmid uptake | | | |
| PF3D7_0525400 | 7-Helix-1-KO integration primers 1 and 2 | TCTTTACGATGGGTGATG | GAGCTGGGAAATAGACTAGAT |
|  | 7-Helix-1-KO integration primers 3 and 4 | TTATTCCTAATCATGTAAATCTTAAA | CAATTAACCCTCACTAAAG |
| PF3D7_0525400 | 7-Helix-1-HA integration primers 1 and 2 | CCGAAGAATCAAAAGGAAAG | AAATAGGTTAGCTAGGGA |
|  | 7-Helix-1-HA integration primers 3 and 4 | AGATAAAATTTGTAGAGA | CAGCGGCATAATCTGGAA |
| PF3D7_0525400 | 7-Helix-1-GFP plasmid detection | agactggatccGATGAAAATAATAATGAT | CAAGTGTTGGCCATGGAA |
| Primers used for quantitative real-time RT-PCR | | | |
| PF3D7_0717700 | Seryl tRNA-ligase | AAGTAGCAGGTCATCGTGGTT | TTCGGCACATTCTTCCATAA |
| PF3D7_1471100 | EXP2 | GGTCACGTATGTGGTGGGTA | TGTGGCAAAGTTGTTTCTGC |
| PF3D7_1343000 | Phosphoethanolamine N-methyltrans-ferase (PMT) | TATCATCCGGAGGTTTGGAA | CCGTGAGTATGTGCTCCGTA |
| PF3D7_0714000 | Histone H2B variant (H2B.Z) | GCTGGAAAAACCTTAGGACCA | TGAAACGGCGTGTTTTGATA |
| PF3D7_0913200 | Elongation factor 1β  (EF-1β) | TTATTCGGTTGGTTTCAAGGA | ATCATCACCTCCTGCAGCTT |
| PF3D7_1204300 | Eukaryotic translation initiation factor 5A (EIF5A) | GCTGGAGCATCACAAACGTA | TTTTGCATGACCATGCTTTC |
| PF3D7_0831900 | Plasmodium exported protein (PHIST), unknown function | ATGGCATGGCGAAGAATTTA | CCTTCCTTTGCAACACCAAT |
| PF3D7_1328700 | Putative ncRNA | ATGGTTAAATTACACTCAACGATTT | TCCTCTCCGTTTTGTTACGG |
| PF3D7_1131100 | SR1 | AACGAGCAATTGGTTATCCCT | TCACAACCGTTCTTGAACCA |
| PF3D7_1215900 | SR10 | TGGTGATATGGAAGGGCAATG | CGTGTCGCCCATCTCAAAAG |
| PF3D7_0422800 | SR12 | CTCATGTGAAGAGCAAGGCC | ACCCAGATCCATTGAATGCA |
| PF3D7_0713400 | SR25 | ATGGGAACTGCTAGCCTTGT | TCTTCTCTTTCTCATTCTCATCACT |
